# Reduced attenuation of neural activation during fear extinction learning in patients with anxiety disorders: a delayed fear extinction fMRI study

**DOI:** 10.1038/s41398-026-04430-7

**Published:** 2026-11-07

**Authors:** Zirong Qian, Yunbo Yang, Jan Richter, Katharina Domschke, Katja Koelkebeck, Jürgen Deckert, Martin J. Herrmann, Jens Plag, Andreas Ströhle, Hans-Ulrich Wittchen, Andre Pittig, Alfons Hamm, Volker Arolt, Udo Dannlowski, Silvia Schneider, Jürgen Margraf, Winfried Rief, Ulrike Lueken, Tilo Kircher, Isabelle C. Ridderbusch, Benjamin Straube

**Affiliations:** 1 Department of Psychiatry and Psychotherapy & Marburg Center for Mind, Brain and Behavior, Philipps-Universität Marburg, Marburg, Germany; 2 Department of Psychiatry and Psychology, Children’s Hospital of Fudan University, Shanghai, People’s Republic of China; 3 Department of Experimental Psychopathology, Institute of Psychology, University of Hildesheim, Hildesheim, Germany; 4 Department of Biological and Clinical Psychology/Psychotherapy, University of Greifswald, Greifswald, Germany; 5 Department of Psychiatry and Psychotherapy, Medical Center-University of Freiburg, Faculty of Medicine, University of Freiburg, Freiburg, Germany; 6 Bielefeld University, Medical School and University Medical Center OWL, Protestant Hospital of the Bethel Foundation, Department of Psychiatry and Psychotherapy, Bielefeld, Germany; 7 Department of Psychiatry, Psychosomatics and Psychotherapy, Center of Mental Health, University Hospital of Würzburg, University of Würzburg, Würzburg, Germany; 8 Institute for Mental Health and Behavioral Medicine, Faculty of Medicine, HMU Health and Medical University Potsdam, Potsdam, Germany; 9 Department of Psychiatry and Psychotherapy, Campus Charité Mitte, Charité –Universitätsmedizin Berlin, Berlin, Germany; 10 Institute of Clinical Psychology and Psychotherapy, Technische Universität Dresden, Dresden, Germany; 11 Department of Psychiatry and Psychotherapy, University Hospital, Ludwig Maximilians University, Munich, Germany; 12 Translational Psychotherapy, Institute of Psychology, Georg-August-Universität Göttingen, Göttingen, Germany; 13 Institute for Translational Psychiatry, University of Münster, Münster, Germany; 14 German Center for Mental Health (DZPG), Site Jena Magdeburg Halle, Halle, Germany; 15 Center for Intervention and Research on Adaptive and Maladaptive Brain Circuits Underlying Mental Health (C-I-R-C), Site Jena Magdeburg Halle, Halle, Germany; 16 Faculty of Psychology, Clinical Child and Adolescent Psychology, Mental Health Research and Treatment Center, Ruhr-Universität Bochum, Bochum, Germany; 17 German Center for Mental Health (DZPG), Site Marburg-Bochum, Bochum, Germany; 18 Mental Health Research and Treatment Center, Faculty of Psychology, Ruhr-Universität Bochum, Bochum, Germany; 19 Department of Clinical Psychology and Psychotherapy, Philipps-Universität Marburg, Marburg, Germany; 20 Department of Psychology, Humboldt-Universität zu Berlin, Berlin, Germany; 21 German Center for Mental Health (DZPG), partner site Berlin-Potsdam, Berlin/Potsdam, Germany

**Keywords:** Neuroscience, Psychiatric disorders

## Abstract

Clarifying the patterns of neural activation during fear extinction in anxiety disorders (ADs) is essential for advancing our understanding of inhibitory learning. However, the underlying mechanisms remain poorly characterized in large, heterogeneous cohorts of patients. Utilizing a delayed fear extinction fMRI paradigm, this study examined neural responses in 280 patients with ADs — comprising panic disorder, agoraphobia, panic disorder with agoraphobia, social anxiety disorder, and multiple specific phobias — compared to 103 healthy controls (HC). The attenuation of neural activation to conditioned fear (CS+) and safety (CS−) stimuli from early versus late extinction was compared between groups. Subjective ratings of US-expectancy, arousal, and valence were also collected. In a whole-brain voxelwise analysis, patients showed significantly reduced attenuation of neural activation across both CS+ and CS− relative to HC from early compared to late phases of extinction learning. This pattern was observed in a broad network, encompassing the insular cortex (IC) and bilateral supplementary motor area (SMA), among other regions. No interaction between group and stimulus type was observed. Region-of-interest analysis of the left anterior insular cortex confirmed that reduced attenuation in this region represents a robust neural signature of altered extinction learning in ADs. Furthermore, patients reported persistently higher fear expectancy and arousal than HC, consistent with sustained alterations in threat-related processing following extinction training. Together, these findings highlight altered extinction-related neural attenuation as a transdiagnostic feature of ADs, offering novel insights into the neural mechanisms underlying fear extinction.

## Introduction

Anxiety disorders (ADs), including panic disorder (PD), agoraphobia (AG), social anxiety disorder (SAD), specific phobias (SP), and generalized anxiety disorder (GAD), are among the most prevalent mental illnesses globally [1]. Extinction learning, the core mechanism underlying exposure therapy [2, 3], involves updating associations such that previously threatening cues (CS+) no longer predict aversive outcomes (US) [4–6]. This process thereby inhibits conditioned fear responses initially elicited by the CS+ [7]. Previous investigations have implicated delayed and reduced extinction as key pathological features of ADs [6, 8–10]. Elucidating the brain regions where activation attenuates after extinction learning is therefore essential for identifying disruptions within the brain’s extinction circuitry and for advancing the mechanistic understanding of ADs.

Neuroimaging research utilizing functional magnetic resonance imaging (fMRI) has long sought to characterize neural alterations in extinction learning across ADs. However, task-based evidence for neural activation during this process remains sparse and inconsistent. In a transdiagnostic sample of patients with SP, GAD, SAD, or PD, no significant group effects in activation were detected during extinction learning, but all anxiety groups exhibited hypoactivation in ventromedial prefrontal cortex (vmPFC) relative to controls when extinction memory was examined the following day [11]. A similar pattern has been observed more broadly across the human fear extinction literature; for example, the amygdala, usually considered central to fear and extinction learning, has shown inconsistent activations during extinction learning across both individual studies and meta-analytic syntheses [12–15]. By contrast, other regions of the extinction circuitry, including the anterior insula and dorsal anterior cingulate cortex (dACC), are more reliably recruited during this process [12, 15]. Such inconsistency may partly reflect the temporally dynamic nature of these neural responses [14], which averaging BOLD signal across an entire extinction phase may fail to resolve.

Dividing the extinction scenario into “early” (initial trials) and “late” phases offers a methodological framework for capturing the subtle functional shifts in neural responsiveness across these distinct phases [5, 14, 16–18], without making any assumptions about the exact linear, quadratic or exponential learning function across the entire extinction period. This phase-level approach is further supported by evidence from animal studies that early and late extinction engage fundamentally distinct neural mechanisms — early extinction is dominated by inhibitory learning via prefrontal-amygdala circuitry, whereas later extinction increasingly involves structural updating of the original fear memory [19]. Converging evidence from human fMRI studies using markedly different analytic approaches supports this phase-level framework. Using multivariate pattern analysis, one study demonstrated that dividing extinction into early and late phases revealed progressive shifts in distributed activation patterns for CS+ across the amygdala, hippocampus, dACC, and vmPFC that were undetectable when BOLD signal was averaged across the entire extinction phase [20]. Separately, dynamic functional connectivity analyses in patients with ADs or post-traumatic stress disorder (PTSD) revealed widespread group differences from healthy controls (HC) during extinction learning that were not detectable when connectivity was averaged across the entire phase, and that were specific to this learning stage [21]. The present study adopts a complementary approach: directly contrasting mean BOLD activation between early and late extinction phases at both whole-brain and predefined region-of-interest (ROI) levels. Empirically, this approach effectively captures the extent of fear reduction, typically exemplified by attenuated neural activity in vigilance-related regions, such as the cingulate gyrus [17], vmPFC and amygdala [5, 16, 22] as extinction learning progresses. In line with this, our pilot study developed and validated this extinction learning paradigm in HC, providing first evidence that BOLD signal decreased from early to late phases of extinction learning across the bilateral anterior insular cortex (AIC), dorsal and rostral part of ACC, IFG, and left hippocampus in response to both CS+ and CS− (CSs) [18]. Using the same paradigm, the present study extends this earlier work by investigating whether and how this attenuation pattern is altered in a substantially larger clinical sample of patients with ADs, compared to a new matched HC group. Such reduced attenuation of neural activation is often characterized by a significantly blunted decrease in BOLD signal from the early compared to the late phase of extinction learning. This pattern may reflect abnormalities in updating the associations between CS+ and the absence of US, potentially signaling the persistence of fear memory associations. Characterizing this phase-dependent attenuation is therefore essential for understanding the neural mechanisms underlying altered extinction in ADs.

Meta-analyses implicate the AIC and vmPFC as critical regions in human fear extinction learning [12, 23]. The AIC, particularly in the right hemisphere, is consistently recruited during late extinction [12], supporting its involvement in safety consolidation. The vmPFC, in contrast, assumes a central function in late extinction and the subsequent retention of extinction memories [17, 24, 25], and is closely associated with the “top-down” modulation of amygdala-driven fear expression [26]. Nevertheless, how these regions shape within-session changes, specifically the differential neural states comparing early and late phases, remains insufficiently delineated in clinical populations.

To address these gaps, we present a rigorously quality-controlled dataset of 280 patients with diverse subtypes of ADs, using a delayed fear extinction fMRI paradigm previously validated within HC [18, 27, 28]. We hypothesized: (1) individuals with ADs would show reduced attenuation of neural activation from early compared to late phases of extinction learning compared to HC, assessed at the whole-brain level; (2) given their established roles in human fear extinction learning, the AIC and vmPFC would additionally show attenuation effects from early to late extinction learning, tested via predefined ROI analyses complementing the primary whole-brain analysis; and (3) this reduced attenuation was expected to prioritize CS+ over CS–, based on evidence that extinction learning-induced changes in functional connectivity (FC) were specific to CS+ in HC [29], although altered CS− processing might also emerge given its clinical relevance [30–32]. Finally, since fear responses are consciously experienced, our extinction training protocol incorporated explicit verbal self-reports for US expectancy, arousal, and valence in response to CSs [33], which were analyzed alongside neural indices to provide a behavioral correlate of extinction learning.

## Methods

### Participants

This study was part of the German National Research Consortium “Providing Tools for Effective Care and Treatment of Anxiety Disorders” (PROTECT-AD), a sub-project within seven research sites investigating the mechanisms and clinical efficacy of exposure-based cognitive behavioral therapy (exposure-based CBT) [34, 35].

Inclusion criteria for this project required patients who: (1) had a primary anxiety disorder diagnosis by DSM-5, including PD, AG, SAD, or multiple SP; (2) were outpatients; (3) aged between 15 and 70 years; (4) scored above 18 points on the HAMA [36] and more than 3 points on the Clinical Global Impressions Scale (CGI) [37]; (5) were able to attend all therapy sessions independently or with accompaniment, and were proficient in the German language. The exclusion criteria were as follows: (1) currently diagnosed with a psychotic disorder, primary mood disorder (such as bipolar I, recurrent or chronic major depression); (2) a substance use disorder (except nicotine) according to the DSM-5; (3) undergoing concomitant psychological or psychiatric treatment; (4) exhibiting acute suicidality; (5) having general medical contraindications; or (6) having a mono-symptomatic specific phobia. In addition, comorbid disorders, such as depressive disorders or other anxiety disorders, were not excluded. Patients receiving psychopharmacological treatment were eligible if they had been on a consistent maintenance regimen for more than three months prior to enrolment. Trained clinical personnel established categorical diagnoses using the standardized and computer-assisted Composite International Diagnostic Interview [38]. The HC group were recruited based on the condition that they had no 12-month history of medical or mental illnesses, as verified by the CIDI. Details of the treatment protocol implemented within the PROTECT-AD consortium are briefly described in the supplementary information (S1). All participants granted written informed consent and the ethical approval was secured from all pertinent institutions.

**Fig. 1:**
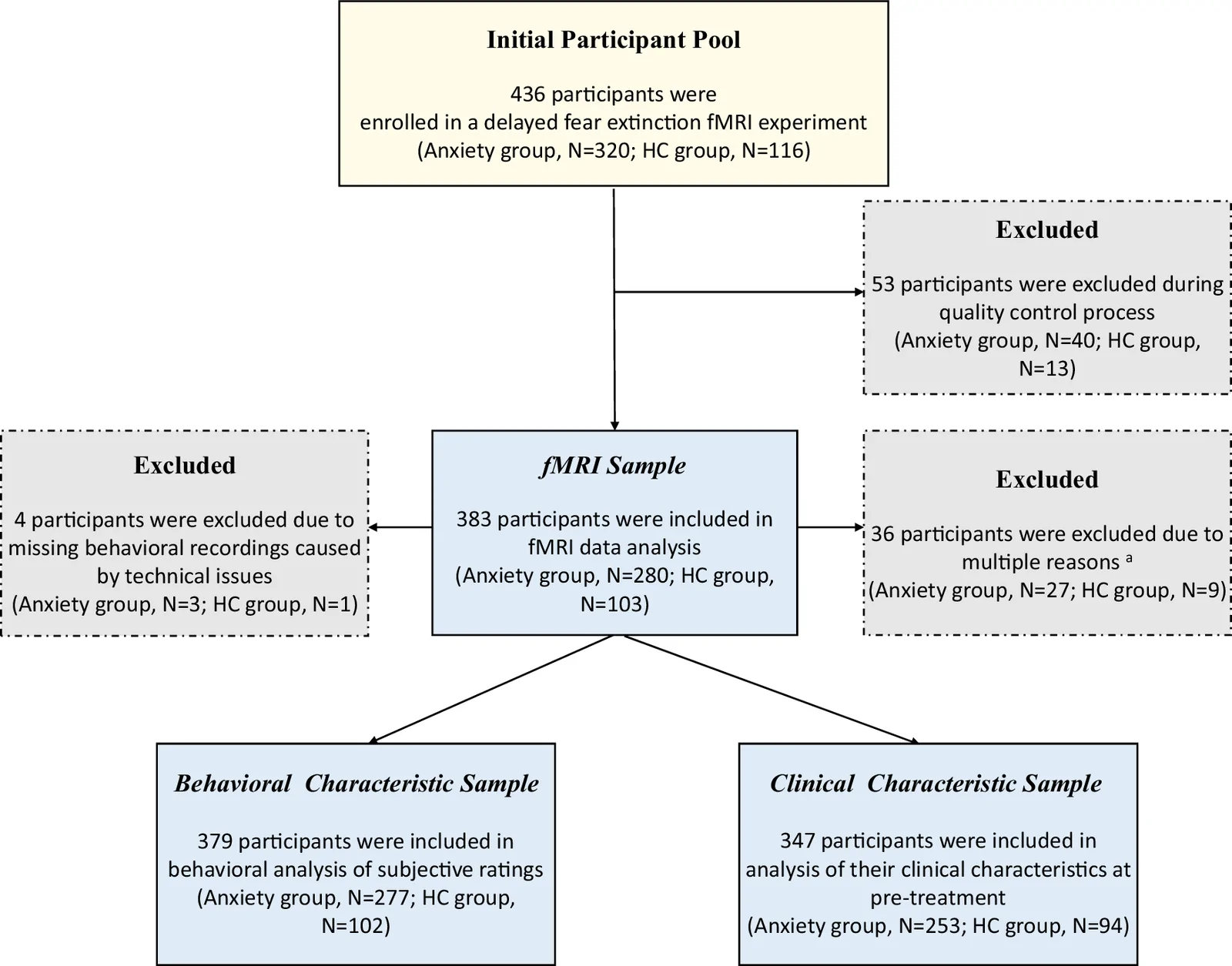
Flowchart of Participant Recruitment and Featured Datasets in the Present Project. ^a^For patients, training patients used to train therapists (N = 25) and pilot patients (N = 2) were excluded. HC were excluded due to missing questionnaire data (N = 9). HC: Healthy Controls.

Figure 1 outlines the flowchart of recruitment pipeline, and clarifies various datasets employed by different analyses. Table 1 showcases demographic information regarding the fMRI sample, along with an overview of the clinical characteristics of the participants at pre-treatment in the clinical characteristic sample.

**Table 1:** Demographic Information of the fMRI Sample and Clinical Information of the Clinical Characteristic Sample.

Demographic characteristics ^a^	Anxiety Group (N = 280)		HC Group (N = 103)		Analysis		
	Mean	SD	Mean	SD	t	df	P
Age (years) ^b^	31.1	10.5	33.3	10.9	1.75	381	0.08
	N	%	N	%	χ²	df	P
Gender ^c^							
Female	145	51.8	49	47.5	0.53	1	0.46
Male	135	48.2	54	52.4			
Research Sites ^d^							
Site 1	67	23.9	16	15.5	11.89	6	0.06
Site 2	2	0.7	0	0			
Site 3	73	26.0	17	16.5			
Site 4	24	8.6	15	14.5			
Site 5	36	12.9	15	14.6			
Site 6	50	17.9	26	25.2			
Site 7	28	10	14	13.5			

Clinical characteristics ^e^	Anxiety Group (N = 253)		HC Group (N = 94)		Analysis		
	N	%	N	%			
Primary Diagnoses ^f^							
	32	12.6					
Agoraphobia	9	3.5					
Panic disorder with agoraphobia	101	40.0					
Social anxiety disorder	96	38.0					
Multiple specific phobia	15	5.9					
	**Mean**	**SD**	**Mean**	**SD**	**t**	**df**	**P**
Measurement ^g^							
HAMA ^h^	24.6	5.6					
ASI ^i^	27.1	10.8	8.6	6.0	−15.7	345	<0.001
BDI ^j^	17.3	10.0	2.1	3.0	−14.6	345	<0.001
PAS ^k^	19.1	10.5					
GAD ^l^	9.2	4.6					
ACQ ^m^	2.0	0.5					
BSQ ^n^	2.4	0.7					
AAQ ^o^	42.0	11.0					

^a^Data represents the fMRI sample.

^b^Age was modeled as a covariate in the fMRI analysis. Anxiety group had a median age of 28 years, ranging from 15 to 67 years. HC group had a median age of 30 years, with a range from 18 to 62 years.

^c^Gender was modeled as a covariate in the main fMRI analysis.

^d^Data was collected from 7 research sites, which was modeled as 7 covariates in the fMRI analysis.

^e^Data represent the clinical characteristic sample.

^f^The procedure for classifying diagnoses was as follows: initial diagnoses were established through the CIDI [61]. This was followed by a thorough clinical evaluation of the CIDI diagnoses conducted by a trained clinician in consultation with the patients. This rigorous process was relevant to the study and treatment within the PROTECT-AD consortium.

^g^Data represent the clinical characteristic sample taken at pre-treatment.

^h^HAMA was developed to assess the severity of anxiety symptoms [36].

^i^ASI was intended to examine the levels of anxiety sensitivity [62].

^j^BDI was implemented to measure characteristic attitudes and symptoms of depression [63].

^k^PAS was utilized to evaluate the severity of panic disorder and agoraphobia [64].

^l^GAD was applied to assess the presence of continuous and uncontrollable worries [65].

^m^ACQ and Body Sensation Questionnaire (BSQ) were designed to measure agoraphobic cognitions and symptom anxiety [66].

^n^AAQ was utilized to evaluate psychological flexibility [67].

^o^*AAQ* = Acceptance and Action Questionnaire, *ACQ* Agoraphobic Cognitions Questionnaire, *ASI* Anxiety sensitivity inventory, *BDI* Beck Depression Inventory, *BSQ* =Body Sensation Questionnaire, *GAD* Generalized Anxiety Disorder, *HAMA* Hamilton Anxiety Scale, *HC* Healthy Controls, *PAS* Panic and Agoraphobia Scale.

### Experimental designs

#### A delayed fear extinction fMRI paradigm

**Fig. 2:**
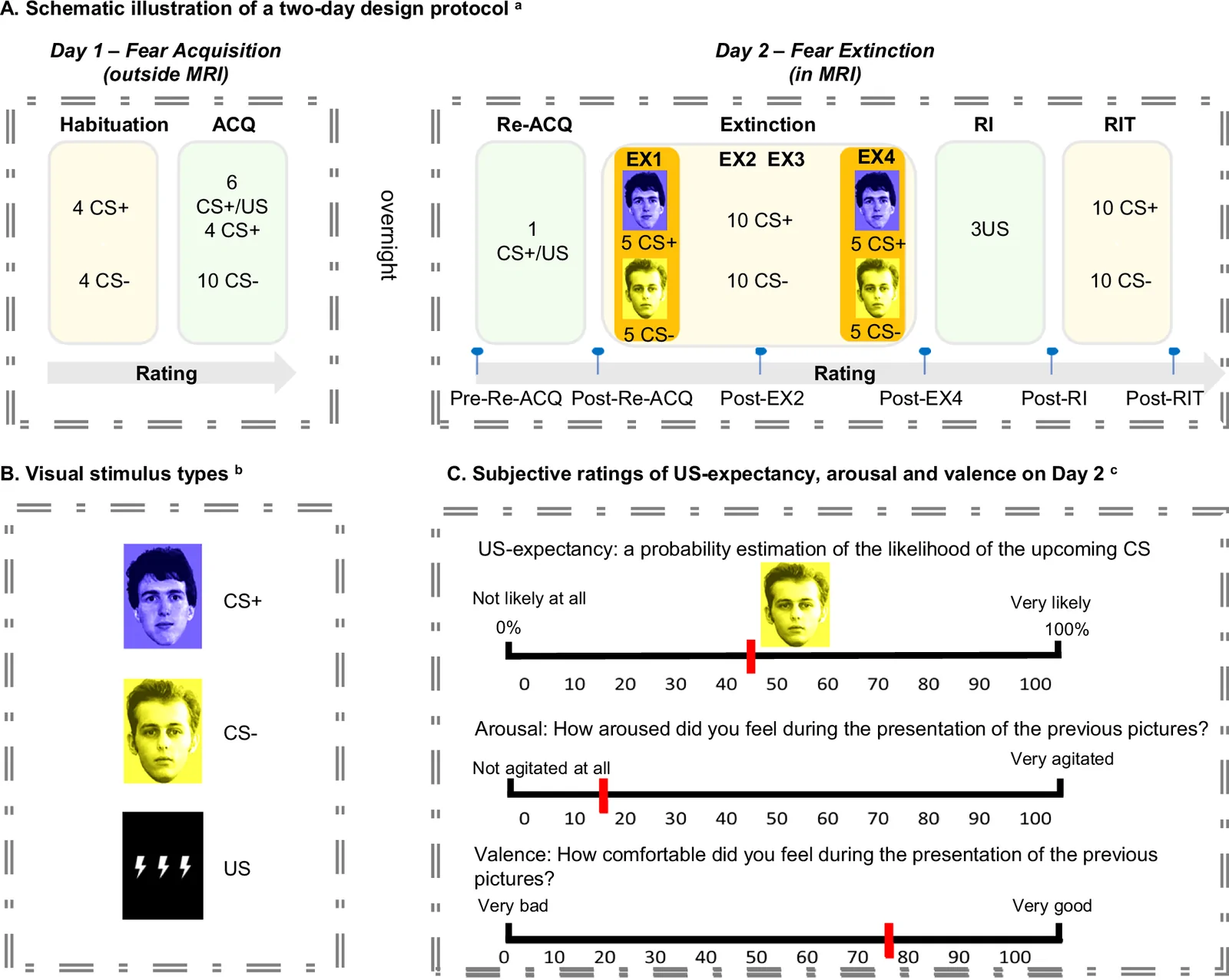
Overview of the Experimental Design, Stimuli, and Subjective Ratings. ^a^Panel **A** illustrates the procedure of a two-day design protocol, that is the procedures of fear acquisition on Day 1 and fear extinction on Day 2. Below the fear extinction, measurement points for subjective ratings of US-expectancy to both CSs are depicted. ^b^Panel **B** presents the stimulus set, including CS+, CS−, and US, used in this study. ^c^Panel **C** shows a diagram illustrating subjective ratings of US-expectancy, arousal and valence in a delayed fear extinction fMRI paradigm. ACQ: Fear Acquisition Block; CS+: Conditioned Fear Stimulus; CS−: Conditioned Safety Stimulus; Extinction: Extinction Block; EX1: Extinction 1; EX2: Extinction 2; EX3: Extinction 3; EX4: Extinction 4; Pre-Re-ACQ: Pre-Fear Reacquisition (before fear reacquisition block); Post-Re-ACQ: Post-Fear Reacquisition (after fear reacquisition block); Post-EX1: Post-Extinction 1; Post-EX2: Post-Extinction 2; Post-RI: Post-Reinstatement; Post-RIT: Post-Reinstatement Test; Re-ACQ: Fear Reacquisition Block; RI: Fear Reinstatement Block; RIT: Fear Reinstatement Test Block; US: Aversive Electric Shock.

A delayed fear extinction fMRI paradigm was embedded in our two-day design protocol [18, 27, 28]. Figure 2a depicts the two-day design protocol [18]: Day 1 involved a differential fear acquisition phase, and Day 2 featured a 24-hour delayed fear extinction phase in the MRI environment. On Day 1, participants underwent an instructed fear acquisition training outside the MRI scanner, where one neutral facial stimulus (designated as CS+) was paired with an aversive electric shock (US) at a 60% rate, while another facial stimulus (CS−) was never paired (as listed in Fig. 2b). On Day 2 (Fig. 2a), the same visual stimuli were used for uninstructed fear extinction. The 24-hour delayed fear extinction fMRI paradigm consisted of four blocks: Re-Acquisition, Extinction, Reinstatement, and Reinstatement Test. As shown in Fig. 2a, the Extinction Block was further divided into four blocks: EX1, EX2, EX3 and EX4, each containing 5 trials per stimulus. In current study, we targeted on EX1 and EX4 (highlighted in orange in Fig. 2a) to identify Early Extinction and Late Extinction respectively on Day 2. The validation of fear acquisition on Day 1 is outlined in the supplement (S2).

#### Subjective ratings

During the fMRI experiment, subjective ratings were measured block-wise using 11-point Likert scales ranging from 0 to 100 (in 10-units increments). The specific dimensions were assessed as follows: (1) US-expectancy: participants estimated the probability that the US would follow the presented CS, with anchors ranging from 0 (“not likely at all”) to 100 (“very likely”); (2) arousal: emotional intensity/alertness ranging from 0 (“not agitated at all”) to 100 (“very agitated”); and (3) valence: emotional response ranging from 0 (“very bad”) to 100 (“very good”) to previous CSs. US-expectancy to both CS+ and CS− was measured at Pre-Re-acquisition Block (Pre-Re-ACQ), Post-Re-Acquisition Block (Post-Re-ACQ), Post-EX2, Post-EX4, Post-Reinstatement Block (Post-RI) and Post-Reinstatement Test (Post-RIT). Subjective ratings of arousal and valence for CS+ were collected at six time points: Post-Re-ACQ, Pre-EX1, Post-EX2, Post-EX4, Post-RI, and Post-RIT; for CS−, ratings were collected at five time points: Pre-EX1, Post-EX2, Post-EX4, Post-RI, and Post-RIT. Figure 2a also shows the block-wise measurement points of US-expectancy during the experiment. Subjective-rating panels for US-expectancy, arousal and valence during experiment are presented in Fig. 2c.

### Data analyses

#### Neuroimaging analyses

##### fMRI data processing

MRI data was acquired using a 3-Tesla MR scanner. Specific MRI parameters and preprocessing pipeline are available in supplement (S3). Preprocessing and analyses of fMRI data were conducted using the Statistical Parametric Mapping (SPM12b) software implemented in MATLAB R2022b (version 9.13; MathWorks). The fMRI analysis implemented a quality control procedure to ensure data quality, as detailed in supplement (S4).

##### Whole-brain voxelwise analysis

We modeled the differential neural activation of early extinction (EX1) versus late extinction (EX4) phases to capture overall magnitude of attenuation in the fitted hemodynamic response. At the first level, a block-wise analysis was conducted for the extinction block (consisting of EX1, EX2, EX3 and EX4), as in previous analyses [28]. To assess the attenuation of neural activity within each block, the 5 trials of CS+ and 5 trials of CS− were modeled as independent regressors to allow for the distinct assessment of neural responses to each stimulus type. We configured a first-level General Linear Model (GLM) that accounted for all images of the paradigm—including all four Extinction blocks, the Reinstatement block, and the Reinstatement Test block—along with six individual head movement parameters per participant as regressors.

To compare the attenuation of neural activation, we established contrast images for each participant based on a common framework where (EX1 > EX4) captures the attenuation of neural activation of early (EX1) versus late (EX4) phases of extinction learning: “attenuation of CS+“ (EX1 CS+> EX4 CS+) and “attenuation of CS−“ (EX1 CS − > EX4 CS−).

In the second-level GLM, whole-brain voxelwise BOLD activity was analyzed using a factorial design (two-way ANCOVA) to evaluate three contrasts of interest: (1) Main effect of group on attenuation: (EX1 > EX4) HC − (EX1 > EX4) Anxiety group, collapsed across CSs, capturing whether HC showed greater overall attenuation of neural activation across stimulus types compared to the anxiety group; this analysis was used to address the first hypothesis; (2) Main effect of stimulus type on attenuation: (CS + EX1 > CS + EX4) − (CS − EX1 > CS − EX4), collapsed across both groups, capturing whether the attenuation of neural activation was greater for CS+ than for CS− across groups; this analysis was used to address the third hypothesis; (3) Group X Stimulus type interaction on attenuation: Group (HC vs. Anxiety group) X Stimulus type (CS+ vs. CS−) interaction on attenuation (EX1 > EX4), capturing whether the differential attenuation between CS+ and CS− differed between HC and the anxiety group.

Age, gender, and research site were included as nuisance covariates to control for potential confounding effects.

Brain regions bearing a significant effect were reported using cluster-level family-wise error (FWE) correction at p  <  0.05. This corrected threshold was achieved by applying a cluster-defining threshold of p  <  0.001 (uncorrected) with continuous 104 voxel (k = 104). This criterion was determined based on a Monte Carlo simulation [39], which was detailed in the supplement (S5). The cluster correction has the advantage of higher sensitivity to smaller effect sizes, while still correcting for multiple comparisons across the whole brain volume [39]. The disadvantage is a reduced specificity regarding smaller clusters, which cannot be detected. Therefore, complementary ROI analyses had been applied.

##### ROI analysis

To test the second hypothesis, the AIC and vmPFC were selected as pre-defined ROIs based on their established roles in human fear extinction learning identified by meta-analysis [12], to further examine whether they exhibited the reduced attenuation of neural activation across the extinction Block, complementing the primary whole-brain analysis. The AIC mask was derived from the SPM Anatomy Toolbox [40], by combining the cytoarchitectonic areas Ia, Id2, Id6, and Ig3 [41]. The vmPFC mask was defined from a meta-analysis on extinction recall [42] (https://identifiers.org/neurovault.image:16258; NeuroVault image ID: 16258), binarized prior to analysis.

At the second-level GLM, the Main effect of group (HC > Anxiety group) was assessed with respect to the attenuation of neural activation from EX1 compared to EX4. Within each ROI, SVC with FWE correction (pFWE-SVC < 0.05) was applied in SPM12b, using an initial voxel-wise threshold of p  <  0.001 (uncorrected) to define clusters.

All neuroimaging analyses were performed on the fMRI sample.

#### Subjective ratings analyses

Given that the Extinction Block is focal to our research, we examined CSs evaluations at three measurement points for each kind of subjective ratings to characterize their overall psychological responses. Specifically, for US-expectancy, we assessed data at Post-Re-ACQ, Post-EX2, Post-EX4 for both CSs; for valence and arousal, we measured data at Pre-EX1, Post-EX2 and Post-EX4. Within-subject effects of time (three measurement points), stimulus (CS+ vs. CS−), and between-subject effects of group (HC vs. Anxiety group) were evaluated by a three-way repeated measures ANOVA, performed in JASP software (Version 0.18.3; JASP Team, 2024). We used the behavioral characteristic sample in these analyses.

### Exploratory analyses

To further characterize the primary finding of reduced neural attenuation from early compared to late extinction in the anxiety group relative to HC, a series of complementary analyses were conducted. First, we examined the global effect of CS + > CS− across EX1 and EX4 in both groups (S6). To delineate which temporal windows drove the observed group differences, neural activation patterns during the Early and Late Extinction phases were examined independently (S7). We further conducted a linear trend analysis across EX1–EX4 to capture the overall trajectory of neural responses throughout the entire extinction trials (S8). Finally, within-group main effects of extinction (EX1 > EX4) were examined separately for the anxiety group and HC to validate the attenuation effects within each group (S9).

To assess the robustness of our primary findings, the main fMRI analysis was replicated in the clinical characteristic sample using identical methodology, with activation patterns — derived from eigenvariates — compared across subgroups of ADs (S10). Behavioral analyses of subjective ratings were conducted using the same covariates as the fMRI models (age, gender, and site) for consistency (S11). We further examined correlations between the magnitude of attenuation to CS+ and behavioral adjustments to CS+ (S12). Finally, between-group comparisons of state anxiety levels at the start of the fMRI session were conducted to assess potential differences in scanner-induced distress; the second-level fMRI analysis was additionally re-run with state anxiety included as a covariate for sensitivity analysis (S13).

The analyses reported in this study were not preregistered.

## Results

### Neuroimaging findings

#### Whole-brain voxelwise analysis

**Fig. 3:**
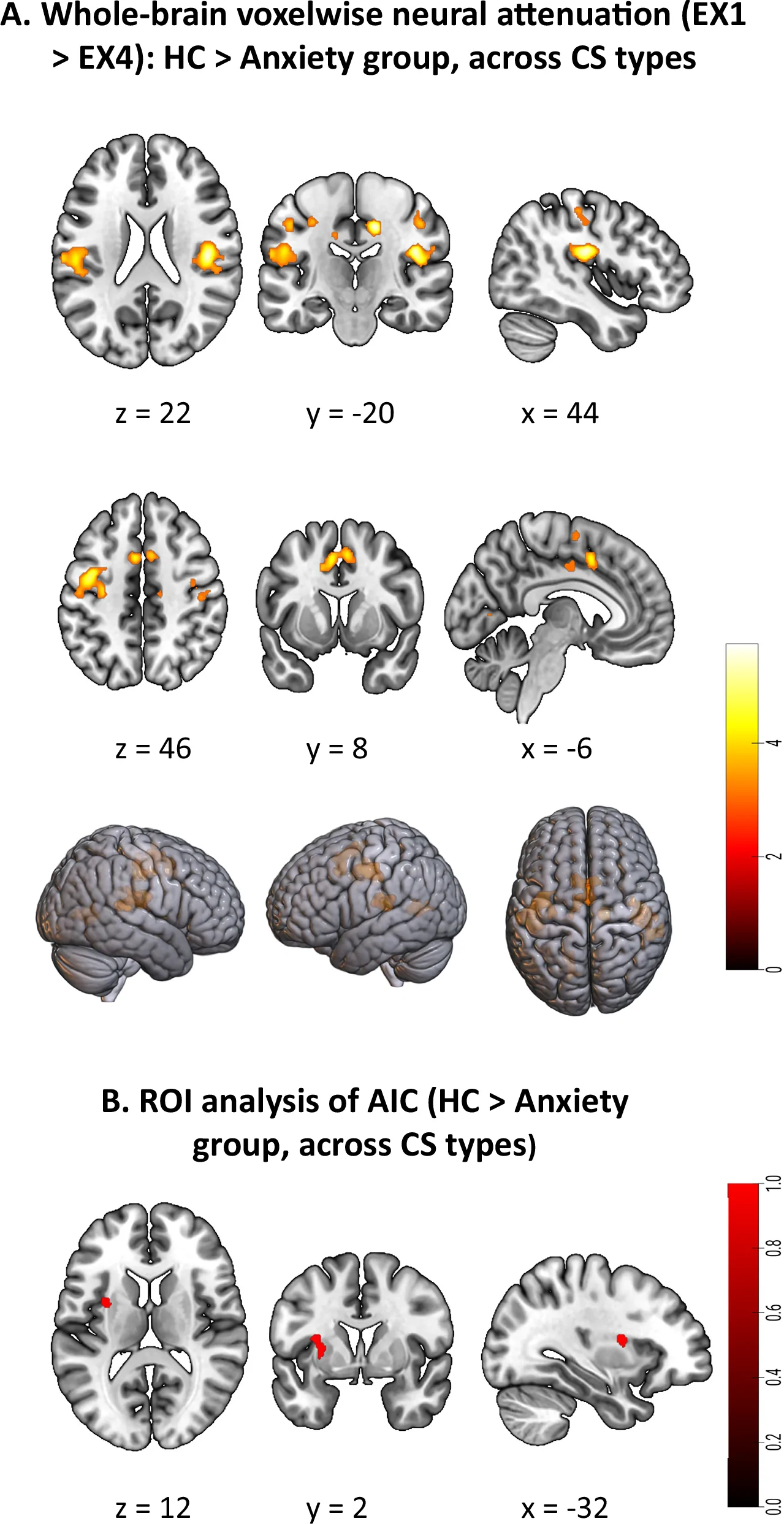
Visualization of Neuroimaging Results. ^a^Panel **A** illustrates the whole-brain voxelwise analysis of main effect of group (HC > Anxiety group) on attenuation, across CS types. Orthogonal slices (axial, coronal, and sagittal) are displayed at two representative peak coordinates identified by this analysis: [40, –20, 22] and [–6, 8, 46]. A 3D brain rendering is provided to show the global distribution of these effects. ^b^Panel **B** shows the ROI analysis of the AIC, illustrating attenuation of neural activation for the contrast HC > Anxiety group (across CS types) above the statistical threshold. HC: Healthy Controls; ROI: Region-of-interest analysis.

**Fig. 4:**
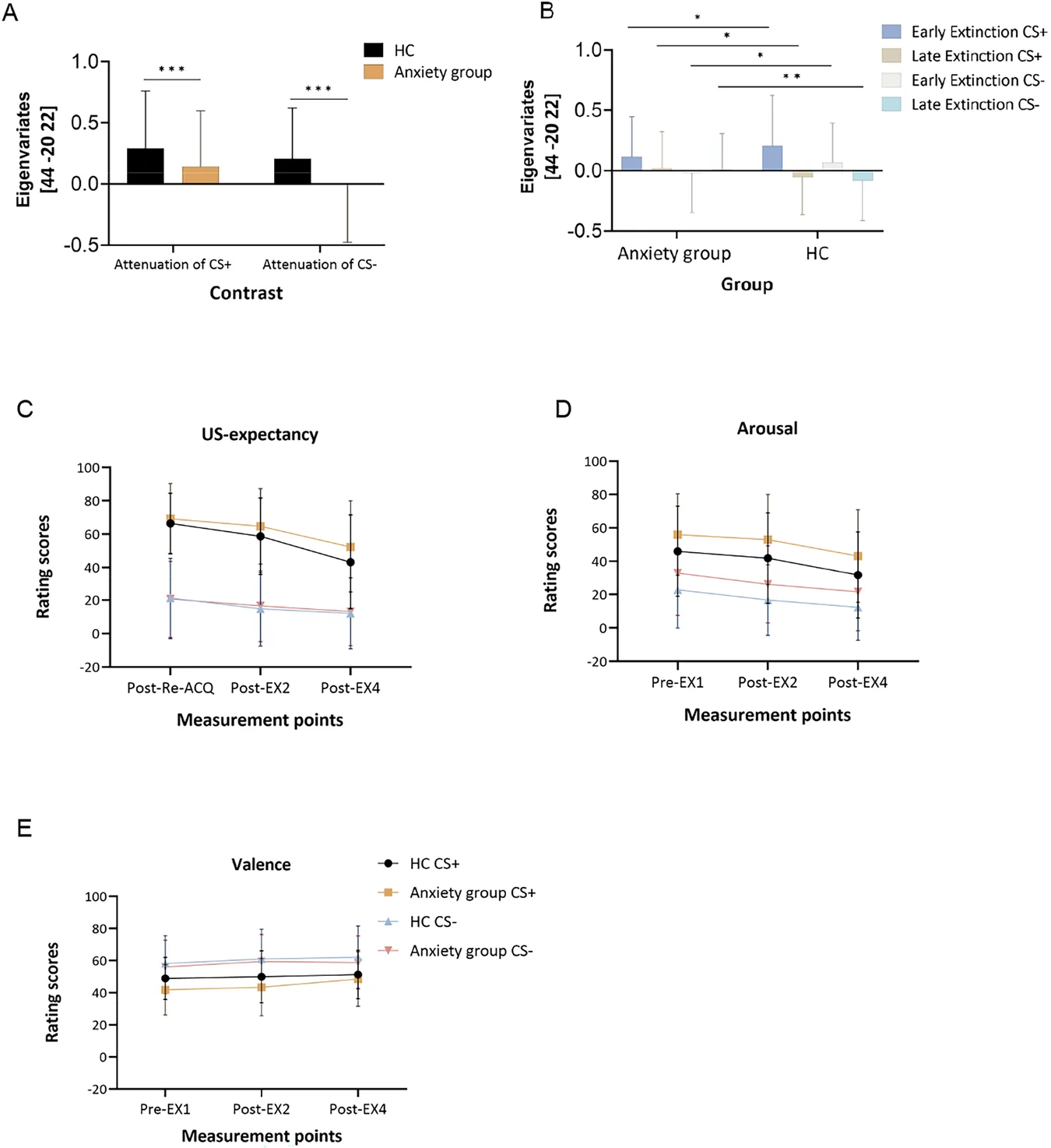
Group Comparisons on magnitude of attenuation (HC> anxiety group) across CSs and Overall Subjective Ratings of US-expectancy, Arousal and Valence. ^a^Panel **A** illustrates a bar chart displaying mean scores and standard deviations of estimated parameters (eigenvariates) of whole-brain voxelwise analysis of main effect of group (HC > Anxiety group, across types). Eigenvariates were obtained by the VOI function of SPM12b in Matlab. ^b^Panel **B** displays a bar chart of distinct activation in Early Extinction CS+, Early Extinction CS−, Late Extinction CS+, Late Extinction CS− respectively between groups respectively, based on the contrast of EX1 > EX4 (HC > anxiety group). ^c^Panel **C,** **D** and **E** represent the mean scores and standard deviations of subjective ratings for US-expectancy, arousal and valence to CSs between groups, respectively. HC: Healthy Controls; Post-EX2: Post-Extinction 2; Post-EX4: Post-Extinction 4; Post-Re-ACQ: Post-Re-acquisition Block; Pre-EX1: Pre-Extinction 1.

Regarding the first hypothesis, a robust main effect of group (HC > anxiety group) was observed, reflected by significantly greater attenuation of neural activation from early versus late extinction in HC compared to the anxiety group [(EX1 > EX4) HC − (EX1 > EX4) Anxiety group], across CSs (Table 2), indicating a significant group X time interaction. As shown in Table 2, a widely distributed set of regional clusters was associated with this effect, encompassing the right IC, bilateral supplementary motor area (SMA), middle cingulate cortex (MCC), extending to adjacent areas. Brain slices of each cluster, based on the peak voxels, are visualized in Fig. 3a. To compare the magnitude of attenuation between groups, we extracted eigenvariates from an 8 mm sphere centered on the most significant peak voxel [44, –20, 22], which also partially included the right posterior insula (see Fig. 4a). However, the reverse contrast [(EX1 > EX4) Anxiety Group − (EX1 > EX4) HC] yielded no significant activation differences across CSs (Table 2).

**Table 2:** Whole-brain analysis results: clusters showing the attenuation of neural activation (EX1 > EX4), probed by the delayed fear extinction fMRI paradigm.

Contrast and Cluster Region ^a^	Side	Cluster Size (voxels)	Peak MNI Coordinates (x, y, z)	Z-value	Cluster p (FWE-corr) ^b^
**[(EX1** > **EX4) HC** − **(EX1** > **EX4) Anxiety group], across CSs**					
**Insula**+ rolandic operculum + Heschl gyrus + precentral gyrus + superior temporal gyrus + supramarginal gyrus	R	525	44, −20, 22	5.56	0.001
Postcentral gyrus + middle frontal gyrus + inferior parietal gyrus (excluding supramarginal and angular gyri) + precentral gyrus	L	471	−38, −2, 48	4.98	0.004
**Supplementary motor area** (R + L) + superior frontal gyrus, medial (L) + middle cingulate & paracingulate gyri (R + L)	R + L	398	−6, 8, 46	4.47	0.004
Postcentral gyrus + supramarginal gyrus + superior temporal gyrus + operculum	L	439	−52, −22, 22	4.29	0.002
Lingual gyrus + precuneus + calcarine	L	213	−18, −58, 4	3.91	0.048
**[(EX1** > **EX4) Anxiety group** − **(EX1** > **EX4) HC], across CSs**					
No differential results					
**[(CS** + **EX1** > **CS** + **EX4)** − **(CS** − **EX1** > **CS** − **EX4)], across groups**					
Supramarginal gyrus + rolandic operculum + superior temporal gyrus	R	211	44, −22, 24	4.32	0.050
Superior temporal gyrus + supramarginal gyrus + postcentral gyrus	L	230	−60, −22, 26	4.31	0.038
**[(CS** − **EX1** > **CS** − **EX4)** − **(CS** + **EX1** > **CS** + **EX4)], across groups**					
No differential results					
**Group (HC vs. Anxiety group) × Stimulus type (CS+ vs. CS**−**) interaction on attenuation (EX1** > **EX4)**					
No differential results					

^a^All contrasts were performed using t-tests; cluster regions were labelled using Automated anatomical labeling (AAL) [68].

^b^Brain regions showing significant effects across all contrasts were reported at an FWE cluster-level correction of p  <  0.05, achieved using a cluster-defining threshold of p < 0.001 (uncorrected) with a minimum cluster size of 104 voxels (k = 104).

^c^*CS* Conditioned Fear Stimulus, *CS* Conditioned Safety Stimulus, CSs: CS+ and CS−*FWE* Family-Wise Error, *HC* Healthy Controls, *L* Left, *MNI* Montreal Neurological Institute, *R* Right.

To further examine the activation patterns during early and late extinction separately, each block was modeled as a single regressor in the second-level GLM, and the same contrast of main effect of group (HC > Anxiety group) on attenuation across CSs was applied. Eigenvariates were extracted from an 8 mm sphere centered around the same peak voxels [44, −20, 22]. Group differences for early extinction and late extinction to CS+ and CS− were then compared separately (see Fig. 4b). The results showed that anxiety group exhibited reduced activation during early extinction to both CS+ and CS− (all p < 0.05).

Taken together, these analyses collectively yielded a significant difference in the magnitude of attenuation from early compared to late phases of extinction training between groups (all p < 0.001), with HC showing greater activation than the anxiety group during the early extinction window for both CSs (all p < 0.05).

Regarding the third hypothesis, greater attenuation of neural activation for CS+ relative to CS− (attenuation of CS + > attenuation of CS−) from early compared to late extinction was observed collapsing across groups [(CS + EX1 > CS + EX4) − (CS − EX1 > CS − EX4)]. As outlined in Table 2, this effect was localized to the right supramarginal gyrus (SMG), left superior temporal gyrus (STG), and other regions. The reverse contrast (attenuation of CS − > attenuation of CS+; [(CS − EX1 > CS − EX4) − (CS + EX1 > CS + EX4)]) did not reach statistical significance, highlighting a differential pattern of neural attenuation favoring CS+ across participants.

Moreover, the group X stimulus interaction effect on attenuation of neural activation, [Group (HC vs. Anxiety group) × Stimulus type (CS+ vs. CS−) interaction on attenuation (EX1 > EX4), Table 2] indicated no significant group-specific differences in the processing of CS+ vs. CS− from early compared to late extinction.

#### Region-of-interest (ROI) analysis

With respect to the second hypothesis, a significant main effect of group (HC > Anxiety group, across CSs) was observed within the pre-defined ROI of AIC, reflecting greater attenuation of neural activation in HC during extinction learning. At the set level, the overall effect within the ROI of AIC was significant (pFWE-SVC = 0.017). Peak-level analysis further revealed a significant cluster in the left AIC (see Fig. 3b; MNI: x = −32, y = 2, z = 12; t = 3.71; k = 8; pFWE-SVC = 0.027), whereas the right AIC showed only a trend-level effect (MNI: x = 34, y = 12, z = 4; t = 3.44; k = 10; pFWE-SVC = 0.070).

These findings indicate that patients exhibited significantly reduced attenuation in the bilateral AIC relative to HC. In contrast, no significant group differences were detected within the predefined ROI of vmPFC.

### Overall Subjective ratings

#### US-expectancy

Figure 4c displays a significant main effect of time (F(2,754) = 129.61, p < 0.001, η^2^ = 0.03), indicating a general adjustment of expectancy across stimuli and groups during extinction training. The main effect of stimulus was significant (F(1,377) = 708.92, p < 0.001, η^2^ = 0.39), with CS+ expectancy consistently higher than CS−. A significant interaction between time and stimulus (F(2,754) = 32.62, p < 0.001, η^2^ = 0.006) was observed, with all post-hoc comparisons being significant (p < 0.001). The main effect of group was significant (F(1,377) = 4.54, p < 0.05, η^2^ = 0.002). No significant interaction effects were found (time X group: F(1,754) = 2.70, p > 0.05: time X stimulus X group: F(1,754) = 1.18, p > 0.05). Post-hoc comparisons showed the anxiety group had higher US-expectancy for CS+ at Post-EX4 than the HC (t = −3.45, p_holm_ < 0.05; p value was adjusted by Bonferroni-Holm Correction).

#### Arousal

As shown in Fig. 4d, there was a significant main effect of time (F(2754) = 80.29, p < 0.001, η^2^ = 0.03), with arousal ratings generally decreasing from Pre-EX1 to Post-EX4 in both groups. A significant main effect of stimulus indicated greater differentiation between CS+ and CS− across both groups and time (F(1, 377) = 278.43, p < 0.001, η^2^ = 0.14). In addition, the anxiety group showed higher arousal ratings compared to the HC (F(1377) = 23.91, p < 0.001, η² = 0.03). However, interactions between time X group, stimulus X group, and time X stimulus X group were not significant (all p > 0.05). Post-hoc analyses revealed that the anxiety group showed greater arousal to CS+ and CS− than HC at Post-EX4 (CS+: t = −3.93, p_holm_ < 0.01; CS−: t = −3.24, p_holm_ < 0.05).

#### Valence

Figure 4e shows that participants rated both CS+ and CS− as more pleasant from Pre-EX1 to Post-EX4, as indicated by a significant main effect of time (F(1,754) = 17.06, p < 0.001, η^2^ = 0.01). A significant main effect of stimulus (F(1, 377) = 111.71, p < 0.001, η^2^ = 0.09) confirmed a clear differentiation between CS+ and CS−. The anxiety group rated CSs more negatively than the HC during extinction training (F(1377) = 11.35, p < 0.001, η^2^ = 0.01). Interactions between time X group, and stimulus X group were not significant (all p > 0.05).

### Exploratory outcomes

The detailed outcomes of the complementary analyses are reported in the supplement contents (S6-S13). Briefly, regarding the neuroimaging findings, the global effects of CS + > CS− across EX1 and EX4 yielded significant activation in the bilateral IC, middle frontal gyrus (MFG), superior frontal gyrus (SFG), and other adjacent regions across both groups; however, no significant group differences (HC > Anxiety group) for CS + > CS− were observed above threshold (S6). Phase-specific analyses suggest that the between-group difference in neural attenuation was predominantly driven by the early extinction phase (S7). A linear trend analysis (EX1 > EX2 > EX3 > EX4) revealed a broad linear decrease in neural activation in HC in response to CS+ during extinction learning (S8). Both the HC and the anxiety group exhibited within-group main effects of neural attenuation (EX1 > EX4) to CSs (S9). The primary neuroimaging findings remained significant and comparable in the clinical characteristic sample. Furthermore, eigenvariates extracted from the ROI mask of the AIC, based on the contrast HC > Anxiety group (across CSs), did not show statistically significant differences across diagnostic subgroups for CSs (all P_tukey_ > 0.05; S10).

Regarding the behavioral analysis, subjective ratings of US-expectancy, arousal, and valence — analyzed using the same covariates as the primary fMRI models (age, gender, and research site) — revealed significant main effects of time, stimulus type and group (all p < 0.05; S11). A significant brain-behavior correlation was observed across both groups, whereby greater attenuation of neural activation in response to CS+ in both a region extending to the right IC (r = 0.11, p < 0.05) and the left SMA (r = 0.11, p < 0.05) was associated with stronger expectation violations for CS+ (S12). Finally, the anxiety group reported significantly higher baseline state anxiety compared to HC (t = −12.705, df = 378, p < 0.001, Cohen’s d = 0.15; S13).

## Discussion

In the present study, we characterized the attenuation of neural activation underlying extinction learning in a large transdiagnostic sample of ADs. Compared with HC, anxiety patients exhibited reduced attenuation of neural activation from early compared to late extinction phases across both CS+ and CS−, particularly within the IC. ROI analyses further indicated that reduced attenuation in the AIC represents a robust neural signature of altered extinction learning in ADs. Across both groups, neural attenuation was more pronounced for CS+ than for CS−, particularly in the bilateral SMG and STG. Subjective ratings mirrored these neurobiological patterns, with anxiety patients persistently reporting higher US-expectancy, arousal, and valence ratings for both CSs throughout extinction learning relative to HC. Together, these findings point to altered patterns of neural attenuation of extinction learning in ADs, suggesting difficulty in flexibly downregulating fear responses and engaging safety learning processes.

Consistent with our pilot study [18], the global effects of CS + > CS− yielded significant activation in the bilateral IC, particularly during early extinction, across both groups (S6 and S7), confirming the robustness of the paradigm in the current larger sample. In line with our first hypothesis, whole-brain voxelwise analysis revealed reduced attenuation of neural activation in anxiety patients relative to HC, extending beyond the posterior part of IC, cingulate cortex to a broader network encompassing regions such as the MCC, SMA, precentral gyrus (PeCG), postcentral gyrus (PoCG), SMG, STG and rolandic operculum — regions widely implicated in extinction learning and threat processing [5, 7, 21, 23, 43–45], consistent with a pain-related conditioned fear circuit [46] likely elicited by electric electrodes as the US in our paradigm. Mechanistically, altered IC downregulation may reflect aberrant integration of aversive signals and an imbalance between extinction and fear memories [47, 48]. The SMA, primarily involved in movement preparation and execution [49], showed rigid activation changes, suggesting compromised suppression of defensive reactions such as avoidance or escape from threatening contexts [5]. This dual dysregulation may underlie the maintenance of hypervigilant psychological states post-threat offset in individuals with ADs. Inhibitory learning is widely regarded as the key mechanism driving extinction processes, though habituation serves as an important prerequisite [3]. Our findings suggest that anxiety patients exhibit a blunted neural profile that may reflect differences in both processes, failing to downregulate their neural responsiveness even to safety signals — rigidity that may impede extinction and hinder consolidation of inhibitory memories. Complementary linear trend analyses across all four extinction blocks (S8) confirmed that group differences reflect a broader linear pattern of attenuation beyond the two selected timepoints.

Following our second hypothesis, the ROI analyses underscored the role of the AIC in extinction-related attenuation, with the HC showing significantly stronger attenuation compared with anxiety patients, representing a focal and robust neural marker of altered extinction learning. In contrast, no significant group differences were observed in the vmPFC, consistent with a large-scale meta-analysis [12] showing vmPFC activation remains inconsistent across task-based fMRI studies, suggesting between-group differences are more heavily driven by threat-related processing within the salience network than prefrontal inhibitory mechanisms. Within-group analyses did reveal significant neural attenuation in vmPFC sub-regions (e.g., pgACC) for both groups (S9-Table 5), confirming vmPFC engagement during extinction learning without significant between-group differences.

In accordance with our third hypothesis, CS−specific analyses revealed that neural downregulation was more pronounced for CS+ than for CS− across both groups, particularly in the SMG and STG. This suggests that processing emotionally charged stimuli requires greater cognitive resources associated with attention, fear anticipation, and memory retrieval — functions linked to the SMG [46, 50]. The STG has previously shown heightened responsiveness to fearful stimuli in healthy populations [51], reinforcing that extinction learning prioritizes threat over safety signals. The IC played a specific role in threat anticipation and executive control [52–56], serving as a key node within the salience network and orchestrating adaptive responses [57, 58], may be the primary driver of extinction-related threat processing in this paradigm.

However, the absence of a group × stimulus interaction on attenuation indicates compromised neural adaptation was not CS+-specific. Phase-specific analyses (Fig. 4b) further revealed that early extinction is the pivotal time window for between-group differences, with anxiety patients showing hypo-activation to CSs relative to HC. This pattern might be plausibly explained by the elevated scanner-induced stress observed in the anxiety group (S13), which could hamper early activation in fear-related regions even prior to task-related modulation, resulting in a generalized reduction in neural responsiveness regardless of stimulus type [25, 59]. This observation harmonizes with our previous findings, where HC with high intolerance of uncertainty (IU) and trait anxiety (TA) demonstrated lower extinction-related activation than HC with low IU/TA [28]. Collectively, these results support the notion that anxiety and anxiety-prone individuals may adopt a “better-safe-than-sorry” strategy during extinction learning [60], leading to more rigid and less adaptable neural responses.

Subjective rating analyses indicated that anxiety patients overestimated contingency and aversiveness throughout the entire extinction training, reflecting neural inflexibility. This persistent harm expectancy could therefore represent a behavioral manifestation of the observed neural rigidity. Moreover, significant correlations between attenuation in BOLD signals to CS+ — observed in regions extending to the right insula and the left SMA — and adjustments in CS+ expectancy ratings further reinforce the brain-behavior link, suggesting that the degree of neural attenuation is directly related to the updating of threat expectations during extinction learning (S12).

Overall, the primary neuroimaging findings demonstrated relative consistency across the sample, with activation patterns replicated in the clinical characteristic sample and no significant differences detected across diagnostic subgroups (S10). These results collectively reinforce the robustness of the observed neural attenuation patterns in ADs. However, this neurobiological consistency was not fully paralleled at the behavioral level, as the group X time interaction did not reach significance in the subjective ratings data. This dissociation between neuroimaging and behavioral measures may suggest that fMRI is more sensitive in capturing subtle differences in neural processing that have not yet manifested at the behavioral level.

Several limitations and future directions warrant consideration. First, the block-based analysis approach offers a coarse estimation of time-dependent BOLD changes; adopting half-blocks analyses may maximize the effect size, although the linear trend analyses across the entire extinction trials revealed a comparable pattern. Subsequent research could further benefit from trial-by-trial analyses to obtain more precise temporal information. Second, beyond behavioral response, incorporating multilevel outcome measures, such as skin conductance responses (SCR) would strengthen the ecological validity of the extinction paradigm. Finally, while the present study adopted a transdiagnostic approach spanning multiple subtypes of ADs, patients with GAD were not represented in the dataset, and future studies should address this gap.

Taken together, the present study highlights reduced attenuation of neural activation in a large, transdiagnostic sample of ADs, advancing our understanding of extinction-related neural processing in this population. By identifying phase-dependent activation patterns as a robust transdiagnostic neural feature across multiple ADs, our findings provide a foundation for future investigations into neural heterogeneity of fear and extinction learning in clinical populations.

## Supplementary information

Supplement

## Data Availability

The data that support the findings of this study are available from the corresponding author upon reasonable request, subject to the data-sharing policy of the PROTECT-AD consortium and applicable data protection regulations.
