# Supplementary material for: Reduced attenuation of neural activation during fear extinction learning in patients with anxiety disorders: a delayed fear extinction fMRI study
Source: Transl Psychiatry. 2026 Nov 7;16:618. doi: 10.1038/s41398-026-04430-7 (PMC13744572; doi:10.1038/s41398-026-04430-7)
Supplement: Supplementary file 1 [file 41398_2026_4430_MOESM1_ESM.docx]

**Supplementary Contents**

# S1. General Design of the Clinical Study Protocol

The detailed protocol for this clinical trial has been comprehensively described in previous publications^1–3^. Here, we briefly describe the medication, treatment sessions, and therapist protocols implemented in our study.

The study protocol allowed for the inclusion of patients with multiple comorbid diagnoses. To avoid confounding effects, patients initiating new or pharmacotherapy for acute anxiety (e.g., SSRIs, benzodiazepines) were excluded. However, patients on maintenance therapy for more than three months were eligible if they still met the inclusion criteria. To ensure study integrity, medication regimens were required to remain consistent and were monitored by trained clinicians throughout the course of treatment. Concurrent psychotherapy was not permitted.

Briefly, patients received 12 primary treatment sessions (Sessions 1–12), each lasting 100 minutes, followed by two booster sessions (Sessions 13-14) conducted at follow-up assessments two and four months after Session 12. The therapeutic content included psychoeducation, functional behavioral analysis, identification of central threat beliefs and maladaptive anxiety control strategies, principles of exposure, the role of prediction error, and the standardized therapeutic exercises.

All therapy sessions were conducted by well-trained clinicians under regular supervision. Treatment integrity was comprehensively assessed by five independent raters blinded to treatment condition. Ratings were established using a randomly selected sample of 350 video recordings, stratified across Sessions 1-14. Additional details are provided in Online Supporting Information of Pittig et al^2^. Overall, treatment integrity and therapist competence ratings were satisfactory.

# S2. Validation of Fear Conditioning Efficacy: Subjective Rating Data from Day 1

To ensure the validity of our fear extinction paradigm on Day 2, it is critical to confirm successful fear conditioning on Day 1. During Day 1, participants completed fear acquisition training outside the MRI scanner, where one neutral facial stimulus (designated as CS+) was paired with an aversive electric shock (US) on 60% of trials, while another neutral facial stimulus (CS−) was never paired with the US. Behavioral data, including subjective ratings of US-expectancy, arousal, and valence, were also collected.

We analyzed the subjective ratings of US-expectancy, arousal, and valence on Day 1, using a subset of 364 participants (healthy controls (HC), N=93; Anxiety group, N=271) from the behavioral characteristic sample on Day 2 (consistent with the main analyses in the manuscript). Data from 15 participants were excluded due to technical issues.

As illustrated in Fig.1, mean scores at each trial during fear acquisition training demonstrate differential responses to CS+ versus CS− between groups. Critically, robust fear conditioning to CS+ was observed across all participants, confirming the validity of the fear extinction paradigm administered on Day 2.

***US-expectancy***

As shown in Fig.1, US-expectancy was measured across two trials for each stimulus type during the pre-fear acquisition phase, followed by 10 trials for each during fear acquisition. A significant increase in US-expectancy for CS+ was observed from the last trial of pre-acquisition (Pre_2) to the first trial of acquisition (Acqu_1), indicating a strong acquisition effect (post-hoc t-test of CS+: t(363) = −28.94, p__holm_ < 0.001). Meanwhile, the expectancy ratings for CS− significantly decreased during this same period (post-hoc t-test of CS−: t(363) = 10.11, p__holm_ < 0.001). This highlights a clear differentiation between the stimuli: participants expected the US to follow CS+ much more than CS− during the transition from the pre-acquisition to the fear acquisition phase. Additionally, during the fear acquisition phase, US-expectancy ratings for CS+ continued to increase overall, while those for CS− showed a gradual decrease.

***Arousal***

Arousal ratings were measured at the end of the pre-acquisition phase and again at the end of the fear acquisition phase. Participants displayed a significant increase in arousal ratings for CS+ from pre-acquisition to the end of fear acquisition, suggesting successful fear learning (t(363) = −16.24, p < 0.001). In contrast, arousal ratings for CS− significantly decreased over the course of the fear conditioning experiment (t(363) = 3.21, p__holm_ < 0.01). These results highlight a divergence in arousal responses: participants became more aroused by CS+ as fear learning progressed, while arousal associated with CS− diminished.

***Valence***

Valence ratings were collected at the end of the pre-acquisition phase and again at the end of the fear acquisition phase. By the end of fear acquisition, participants rated CS+ as significantly more negative (t(363) = 9.54, p__holm_ < 0.001), consistent with the successful induction of fear conditioning. In contrast, participants rated CS− as significantly more positive (t(363) = −8.33, p__holm_ < 0.001).

Collectively, these findings demonstrate robust and successful fear conditioning across both groups, as evidenced by distinct CS+ versus CS− differentiation in US-expectancy, arousal, and valence ratings.

# S3. fMRI Acquisition Specifications and Image Preprocessing Procedures

As documented in our previous studies^4–6^, we summarize key parameters here. Data were collected using 3-Tesla MR scanners (3× Siemens TrioTim, 1× Siemens Verio, 1× Siemens Prisma, 1× Siemens SKyra) with a 12-channel head coil. Functional images were obtained using a T2-weighted gradient-echo echo-planar imaging (EPI) sequence sensitive to BOLD contrast (TE = 30 ms, TR = 2000 ms, flip angle 90°, matrix size 64 × 64 voxels, voxel size 3.6 × 3.6 × 4.0 mm, slice thickness 4.0 mm, interslice gap 0.4 mm, field of view [FOV] = 230 mm, 33 slices, ascending phase encoding direction). Due to technical limitations, TE = 29 ms was used on the Siemens Prisma, and only 31 slices were recorded on the Siemens Verio. A total of 590 volumes were collected. High-resolution T1-weighted anatomical images were also acquired, as detailed previously^7^. The preprocessing pipeline comprised co-registration of functional images to the individual's high-resolution T1-weighted anatomical image, spatial realignment, normalization to the Montreal Neurological Institute (MNI) template, and spatial smoothing using an 8 mm isotropic Gaussian filter^8^.

# S4. fMRI Data Quality Assessment

A total of 436 participants were enrolled in the fMRI fear extinction paradigm (HC, N = 116; Anxiety group, N = 320). Participants were excluded based on the following MRI data quality criteria: (1) translation or head movement exceeding 1.5 voxel sizes (6 mm), resulting in the exclusion of 3 HC and 10 patients; (2) percent signal change (PSC) exceeding 2.5 *SD* above the mean^9^, resulting in the exclusion of 2 HC and 5 patients; and (3) visual inspection revealing artifacts, resulting in the exclusion of 8 HC and 25 patients. In total, 40 patients (12.5% of the initial patient pool) and 13 HC (11.2% of the initial HC pool) were excluded following quality control procedures. Consequently, 383 participants (HC, N = 103; Anxiety group, N = 280) were retained and included in the final fMRI analyses.

# S5. Cluster-Extent Threshold Determination via Monte Carlo Simulation

The cluster-extent threshold for the fMRI analyses was determined using a custom Monte Carlo simulation ^10^ implemented in MATLAB R2022b (version 9.13; MathWorks, Natick, MA, USA). The simulation was based on the image dimensions (64×64×33 voxels, voxel size 2×2×2 mm³), the estimated spatial smoothness of the residuals (FWHM = 11 mm), and 10,000 iterations. The cluster-level family-wise error rate (FWE) was set at α = 0.05, with voxel-level p-thresholds of 0.001. The simulation assumed Gaussian-distributed noise and no explicit whole-brain mask. For the analyses reported in this manuscript, we used a voxel-level p-threshold of 0.001, for which the simulation yielded a minimum cluster size of 104 voxels (2×2×2 mm³ each; total volume = 832 mm³, i.e., 0.832 cm³).

# S6. Global effect of CS+ > CS− Across EX1 and EX4

As supplementary information for the primary fMRI analysis examining the attenuation of neural activation from EX1 compared to EX4, we further explored the global effect of CS+ > CS− across EX1 and EX4. We applied the same threshold as in the primary analysis, namely, a cluster-defining threshold of p < 0.001 (uncorrected) with k = 104 voxels (FWE cluster corrected).

As shown in Table 1, the global effect of CS+ > CS− across both groups yielded significant activation in the bilateral insular cortex, middle frontal gyrus, superior frontal gyrus, among other regions. No significant activation clusters exceeding the threshold were observed for the group differences (HC > Anxiety group) for CS+ > CS−.

# S7. Phase-Specific Analyses of Neural Activation in Early Extinction or Late Extinction

To delineate which temporal windows contribute to the group differences observed in our primary results (i.e., the reduced attenuation of neural activation from early versus late extinction in the anxiety group compared to the HC), we independently examined neural activation patterns during the early and late extinction phases (Table 2). This supplemental analysis aimed to isolate the phase-specific drivers of the observed neural attenuation profiles.

As detailed in Table 2, the early extinction phase was characterized by a widespread pattern wherein the anxiety group was less activated than healthy controls (HC > Anxiety group) across both CS+ and CS− (see Early Extinction: HC > Anxiety group (across CSs) in Table 2). This divergence was localized in several functionally distinct clusters, encompassing the bilateral lingual gyrus, cuneus, calcarine, and superior/inferior occipital gyri, superior temporal gyrus (STG), precuneus, supplementary motor area (SMA), and middle cingulate gyrus. In contrast, during the late extinction phase, the regions showing significantly less activation in the anxiety group were notably more circumscribed, primarily involving the bilateral lingual gyrus, calcarine sulcus, and fusiform gyrus.

# Together, these phase-specific findings clarify our primary results, suggesting that the differential attenuation of neural activation between groups is predominantly driven by the early extinction phase.

# S8. Linear Trend Analysis Across EX1, EX2, EX3, and EX4

In the primary fMRI analysis reported in the manuscript, we compared the EX1 > EX4 contrast to assess the attenuation of neural activation from early versus late phases of extinction learning. To complement this, we conducted a linear trend analysis across EX1, EX2, EX3, and EX4. To do so, we configured a first-level General Linear Model (GLM) for each participant, modeling the CS+ and CS− trials for each extinction block separately, along with six head movement parameters as regressors. To evaluate the linear decrease in neural activation, a linear contrast [3, 1, −1, −3] was applied to [EX1, EX2, EX3, EX4] at the second-level GLM, respectively, to capture the linearly declining activity across the four extinction blocks for both the CS+ and CS− conditions. Whole-brain voxelwise BOLD activity was then analyzed using a two-way ANCOVA to evaluate the main effects of Group (HC vs. Anxiety group) and CS−specific linear decrease (attenuation of CS+ vs. attenuation of CS−), as well as their interaction. Covariates of no interest (age, gender, and research site) were incorporated into the design matrix. The same threshold as in the primary analysis was applied (p < 0.001 uncorrected; k = 104 voxels).

As shown in Table 3, the HC group demonstrated a linear decrease in neural activation at [44, −16, 24] (HC > Anxiety group). This cluster included the right Rolandic operculum and supramarginal gyrus and extended to the right posterior insula. To visualize the declining trajectory, eigenvariates were extracted from a sphere of 8 mm radius centered on [44, −16, 24]. As shown in Fig.2, the HC group exhibited a linear decreasing trajectory in response to CS+ across extinction blocks.

These converging findings across both contrast weight specifications confirm the robustness of the observed group difference in extinction-related attenuation of neural activation.

# S9. Within-group Main Effects of Attenuation of Neural Activation

To validate the experimental paradigm and facilitate comparison with our previous pilot work, we examined the within-group main effects of neural attenuation (EX1 > EX4) for both the HC and anxiety group. The detailed results are displayed in Table 4. Consistent with our pilot study, the current results replicated the patterns of neural attenuation across both groups. Specifically, both the HC and the anxiety group exhibited significant clusters of decreasing activation in response to CSs during extinction learning. Key regions of attenuation included the insula, anterior cingulate cortex (dorsal and rostral subdivisions), and inferior frontal gyrus.

These findings confirm that the extinction task successfully engaged the expected neural networks in both groups. Furthermore, the results allow for a direct comparison, showing that while the primary task effect is present in both groups, the magnitude and extent of these attenuations differed between groups, consistent with the primary analyses.

# S10. Examination of the Clinical Characteristic Sample

To assess the reliability of the primary neuroimaging findings — which demonstrated that patients exhibit reduced attenuation of neural activation from early to late extinction phases compared to HC — we conducted additional analyses using a more narrowly defined clinical subset (referred to as the “clinical characteristic sample”; see Fig.1 of the manuscript). Specifically, we compared the Anxiety group (N = 253) and HC group (N = 103).

Using analysis methods identical to those described in the whole-brain voxelwise analysis section of the Methods in the manuscript, the results showed strong consistency with the main outcomes, suggesting the robustness and stability of the primary neuroimaging patterns across different samples.

***Reduced attenuation of neural activation from early extinction versus late extinction***

A significant main effect of group was identified (HC > Anxiety group), revealing reduced neural attenuation in the anxiety group relative to HC across both stimulus types. This effect was pronounced within clusters extending to the right insula, Rolandic operculum, STG and supramarginal gyrus. In the left hemisphere, significant group differences were observed in the superior frontal gyrus, SMA, and STG. The opposite contrast (Anxiety group > HC) did not reach statistical significance, further highlighting the less pronounced attenuation of neural activation in the anxiety group during extinction learning.

Regarding stimulus effects (attenuation of CS+ vs. attenuation of CS−), greater attenuation for CS+ compared to CS− was observed across both groups, localized within the left postcentral gyrus and bilateral SMG. Conversely, the opposite pattern (attenuation of CS− > attenuation of CS+) was significant in regions including the inferior and middle occipital gyrus. Consistent with the main manuscript, no significant interaction was observed between group and stimulus effects on attenuation.

***Subgroup-level analyses***

To further examine potential diagnostic specificity, we conducted a follow-up analysis based on the Region-of-Interest (ROI) results of the AIC reported in the primary manuscript. Specifically, we extracted the first eigenvariate from the AIC ROI mask to compare attenuation of neural activation across diagnostic groups (agoraphobia, panic disorder, panic disorder with agoraphobia, social anxiety disorder, and multiple specific phobia).

An ANOVA was performed with diagnostic subgroup as the between-subjects factor and the first eigenvariate of neural attenuation in ROI of AIC as the dependent variable. For the magnitude of CS+ attenuation, no significant differences were observed across diagnostic categories within the AIC (F = 0.586, df = 4, p > 0.05). Post-hoc pairwise comparisons between diagnostic groups were also not significant (all p__tukey_ > 0.05). Similarly, for the CS− attenuation, the subgroup effect did not reach statistical significance (F = 2.153, df = 4, p > 0.05), and all post-hoc tests were non-significant (all p__tukey_ > 0.05).

Within the framework of null-hypothesis significance testing (NHST), it should be noted that these non-significant results indicate an absence of evidence for group differences, rather than evidence of equivalence. Nonetheless, the consistent lack of diagnostic-specific effects suggests that reduced attenuation of neural activation in the AIC may represent a shared transdiagnostic signature across various anxiety disorders, rather than being limited to any single diagnostic subtype.

***Sensitivity analysis: control for medication effects***

To examine whether the observed neural activation patterns were influenced by psychotropic medication, we conducted a sensitivity analysis on the anxiety group. We extracted the eigenvariates from the significant peak voxel identified in the main effect of group (HC > Anxiety group; MNI coordinates: [44, –20, 22]).

Within the anxiety group, we performed an independent samples t-test to compare the brain responses (eigenvariates for attenuation of CS+ and CS−) between patients who were receiving psychiatric medication at baseline (N = 67) and those who were not (N = 178). Patients with missing medication data (N = 35) were excluded from this analysis.

No significant differences in brain responses were observed between medicated and non-medicated subgroups (attenuation of CS+: t = 1.09, df = 121.02, p > 0.05; attenuation of CS−: t = −0.46, df = 125.84, p > 0.05). These results suggest that the observed brain responses are robust and not primarily driven by psychotropic medication, and that baseline medication status does not appear to be a significant confounder in our primary findings.

# S11. Subjective Ratings Analyses on Day 2

To harmonize the analytical pipeline with our fMRI data, we conducted supplementary analyses on subjective ratings using the same covariates (age, gender, and research site) as employed in the primary neuroimaging analyses. Due to an insufficient number of participants for robust model estimation, Site 2 was excluded from these specific models.

We performed repeated measures ANOVA for US-expectancy, arousal, and valence, respectively. For each model, the within-subject factors included Time (three measurement points) and Stimulus Type (CS+ vs. CS−), while the between-subject factor was Group (Anxiety group vs. HC). Age, gender, and research site (Sites 1, 3–7) were included as covariates to control for confounding variance; their individual effects were not further interpreted.

The results revealed significant main effects of Time, Stimulus, and Group for all three measures (all p < 0.05; see Tables 5, 6, and 7). These findings closely mirror the patterns observed in the primary behavioral analyses reported in the main manuscript, further confirming the robustness of the behavioral effects when accounting for site-related and demographic variance.

# S12. Brain-Behavior Correlation: Neural Activation and Psychological Responding

To investigate whether the observed neural attenuation reflects meaningful changes in psychological responding, we performed post-hoc correlation analyses using the behavioral characteristic sample (N = 379, defined in Fig.1 of the manuscript). Specifically, we examined the relationship between the magnitude of CS+ attenuation and the corresponding changes in US-expectancy ratings for the CS+.

Neural data were represented by the first eigenvariates extracted from the respective clusters with the two peak coordinates: [44, −20, 22] (encompassing parts of the posterior insula) and [−6, 8, 46] (encompassing the left supplementary motor cortex). These coordinates were derived from the contrast of main effect of group (HC > Anxiety group) across CSs regarding the attenuation of neural activation. The adjustment in CS+ expectancy was calculated as the absolute difference in rating scores between the pre-extinction (Post-Re-ACQ) and post-extinction (Post-EX4) assessments.

A significant brain-behavior correlation was observed across the entire sample. Greater attenuation of neural activation in response to the CS+ in both selected regions was significantly associated with a more pronounced decline in CS+ expectancy ratings ([44, −20, 22]: r = 0.11, p < 0.05; [−6, 8, 46]: r = 0.11, p < 0.05). These findings suggest a coupling between neural and behavioral updating during extinction learning.

**S13. State anxiety in the Scanner**

We assessed state anxiety as an index of scanner-related distress at baseline prior to the fMRI fear extinction session, comparing the HC group and anxiety group. An independent t-test was conducted to evaluate differences in state anxiety levels between the two groups.

A total of 380 participants were included in the analysis (HC, N = 102; Anxiety group, N = 278); data for 3 participants were excluded due to missing state anxiety recordings at baseline. The mean and standard deviation for state anxiety were 0.88 ± 1.54 for the HC group and 4.32 ± 2.56 for the anxiety group.

The independent t-test revealed that the anxiety group reported significantly higher baseline state anxiety than the HC group (t = −12.705, df = 378, p < 0.001, Cohen’s d = 0.15).

***Sensitivity Analysis***

As a sensitivity analysis, we re-ran the second-level fMRI analysis including state anxiety as a covariate. For the contrast HC > Anxiety group regarding the attenuation of neural activation across CSs, the results were highly consistent with those reported in the primary analysis, with key clusters remaining significant (see Table 8). These findings indicate that although baseline scanner-related state anxiety differs substantially between groups, adjusting for this factor does not materially alter the primary result: the anxiety group exhibited reduced attenuation of neural activation compared to HC during extinction training.

**Figures and Tables**

**Fig.1** The mean scores and standard deviations of the subjective ratings of US-expectancy, arousal, and valence to CS+ and CS− on Day 1 between two groups.

Acqu_1: fear acquisition 1; Acqu_10: fear acquisition 10; Acqu_2: fear acquisition 2; Acqu_3: fear acquisition 2; Acqu_4: fear acquisition 4; Acqu_5: fear acquisition 5; Acqu_6: fear acquisition 6; Acqu_7: fear acquisition 7; Acqu_8: fear acquisition 8; Acqu_9: fear acquisition 9; Acqu_end: the end of the fear acquisition; CS+: conditioned fearful stimulus; CS−: conditioned safety stimulus; Pre_1: Pre-fear acquisition 1; Pre_2: Pre-fear acquisition 2; Pre_end: the end of the pre-fear acquisition.

**Fig 2.** Linear decrease analysis for the contrast of HC > Anxiety group (across CSs).

CS+: conditioned fearful stimulus; CS−: conditioned safety stimulus; EX1: Extinction 1; EX2: Extinction 2; EX3: Extinction 3; EX4: Extinction 4; HC: Healthy controls.

| **Table 1.** Whole-brain analysis results: clusters showing the general activation across Early (EX1) and Late (EX4) Extinction, probed by the delayed fear extinction fMRI paradigm | | | | | |
| --- | --- | --- | --- | --- | --- |
| **Contrast and Cluster Region** | **Side** | **no. voxels** | **MNI coordinates**  **peak voxel**  **(x, y, z)** | **Z-value** | **Cluster p**  **(FWE-corr)** |
| **CS+ > CS− (across groups)** | | | | | |
| Precentral gyrus (R+L) + insula (R+L) + caudate (R+L) + lenticular nucleus, pallidum (R+L) + inferior frontal gyrus, triangular part (R+L) + inferior frontal gyrus, opercular part (R+L) + IFG pars orbitalis (R+L) + putamen (R+L) + superior temporal gyrus (R+L) + middle frontal gyrus (R+L) + rolandic operculum (R+L) + posterior orbital gyrus (R+L) + thalamus (R+L) | R+L | 7646 | 34, 24, 0 | Inf | < 0.001 |
| Superior frontal gyrus, medial (R+L) + middle cingulate & paracingulate gyri (R+L) + anterior cingulate & paracingulate gyri (R+L) + supplementary motor area (R+L) + superior frontal gyrus, dorsolateral (R) | R+L | 5309 | 6, 38, 40 | Inf | < 0.001 |
| Angular gyrus + middle temporal gyrus + supramarginal gyrus + insula + rolandic operculum + putamen + postcentral gyrus + superior parietal gyrus + superior temporal gyrus + inferior parietal gyrus, excluding supramarginal and angular gyri + Heschl’s gyrus | R | 2861 | 60, -42, 32 | Inf | < 0.001 |
| Angular gyrus+ middle temporal gyrus + supramarginal gyrus + inferior parietal gyrus, excluding supramarginal and angular gyri + superior temporal gyrus + postcentral gyrus + rolandic operculum | L | 1465 | -58, -26, 24 | Inf | < 0.001 |
| Middle frontal gyrus + superior frontal gyrus, dorsolateral | R | 276 | 30, 50, 20 | 4.53 | 0.016 |
| Inferior frontal gyrus, triangular part + middle frontal gyrus + superior frontal gyrus, dorsolateral | L | 443 | -35, 44, 20 | 4.48 | 0.002 |
| **CS+ > CS− (HC > Anxiety group)** | | | | | |
| No differential results |  |  |  |  |  |
| **Abbreviations:** CS+: conditioned fearful stimulus; CS−: conditioned safety stimulus; HC: healthy controls; L: left; MNI: Montreal Neurological Institute; no. voxel: number of voxels per cluster; R: right. | | | | | |

| **Table 2.** Whole-brain analysis results: clusters showing the activation in (EX1) or Late (EX4) Extinction, probed by the delayed fear extinction fMRI paradigm | | | | | |
| --- | --- | --- | --- | --- | --- |
| **Contrast and Cluster Region** | **Side** | **no. voxels** | **MNI coordinates**  **peak voxel**  **(x, y, z)** | **Z-value** | **Cluster p**  **(FWE-corr)** |
| **Early Extinction: CS+ > CS− (across groups)** | | | | | |
| Postcentral gyrus (L) + insula (R+L) + caudate (R+L) + inferior frontal gyrus, opercular part (R+L) + inferior frontal gyrus, triangular part (R+L) + pallidum (R+L) + IFG pars orbitalis (R+L) + posterior orbital gyrus (R+L) + rolandic operculum (R+L) + superior temporal gyrus (R+L) + putamen (R+L) + temporal pole: superior temporal gyrus (R) | R+L | 6745 | 34, 24, 4 | Inf | < 0.001 |
| Superior temporal gyrus + middle temporal gyrus + inferior parietal gyrus, excluding supramarginal and angular gyri + supramarginal gyrus + postcentral gyrus + rolandic operculum | L | 1481 | -58, -24, 24 | Inf | < 0.001 |
| Angular gyrus + middle temporal gyrus + insula + rolandic operculum + supramarginal gyrus + postcentral gyrus + inferior parietal gyrus, excluding supramarginal and angular gyri + putamen + superior temporal gyrus + Heschl gyrus | R | 2065 | 52, -24, 22 | 6.94 | < 0.001 |
| Superior frontal gyrus, medial (R+L) + supplementary motor area (R+L) + middle cingulate & paracingulate gyri (R+L) + anterior cingulate & paracingulate gyri (R+L) + superior frontal gyrus, medial (R) + superior frontal gyrus, dorsolateral (R+L) | R | 4725 | 2, 16, 26 | 6.76 | < 0.001 |
| Middle temporal gyrus + superior temporal gyrus | R | 249 | 50, -28, -6 | 5.25 | 0.024 |
| Superior frontal gyrus, dorsolateral + middle frontal gyrus + postcentral gyrus | R | 282 | 44, -2, 54 | 5.07 | 0.015 |
| **Early Extinction: CS+ > CS− (HC > Anxiety group)** | | | | | |
| No differential results |  |  |  |  |  |
| **Early Extinction: HC > Anxiety group (across CSs)** | | | | | |
| Lobule VI of cerebellar hemisphere (L) + lingual gyrus (R+L) + cuneus (R+L) + calcarine (R+L) + precuneus (R+L) + superior occipital gyrus (R) + inferior occipital gyrus (L) + fusiform gyrus (L) | R+L | 3092 | 12, -66, 8 | 5.17 | < 0.001 |
| Angular gyrus + superior temporal gyrus + supramarginal gyrus + inferior parietal gyrus, excluding supramarginal and angular gyri | R | 357 | 52, -46, 28 | 5.07 | < 0.001 |
| Precuneus (R) + supplementary motor area (R+L) + middle cingulate & paracingulate gyri (R+L) + paracentral lobule (R) | R+L | 935 | -10, -8, 42 | 4.67 | < 0.001 |
| Superior temporal gyrus + supramarginal gyrus + rolandic operculum | R | 217 | 46, -28, 24 | 4.50 | < 0.001 |
| **Late extinction: CS+ > CS− (across groups)** | | | | | |
| Putamen + IFG pars orbitalis + inferior frontal gyrus, triangular part + inferior frontal gyrus, opercular part + rolandic operculum + posterior orbital gyrus + insula | R | 1609 | 34, 24, -2 | Inf | < 0.001 |
| Putamen + IFG pars orbitalis + rolandic operculum + inferior frontal gyrus, opercular part + inferior frontal gyrus, triangular part + temporal pole: superior temporal gyrus + insula + posterior orbital gyrus | L | 1314 | -30, 22, -4 | Inf | < 0.001 |
| Superior frontal gyrus, medial (R+L) + anterior cingulate & paracingulate gyri (R+L) + middle cingulate & paracingulate gyri (R+L) + supplementary motor area (R+L) + superior frontal gyrus, dorsolateral (R) | R+L | 1738 | 6, 36, 40 | 6.42 | < 0.001 |
| Angular gyrus + supramarginal gyrus + superior temporal gyrus + rolandic operculum + inferior parietal gyrus, excluding supramarginal and angular gyri | R | 822 | 60, -44, 32 | 5.86 | < 0.001 |
| Middle temporal gyrus + superior temporal gyrus | R | 311 | 56, -30, -6 | 5.54 | 0.010 |
| Angular gyrus + supramarginal gyrus + inferior parietal gyrus, excluding supramarginal and angular gyri | L | 439 | -58, -50, 32 | 5.44 | 0.002 |
| Middle cingulate & paracingulate gyri (R+L) | R+L | 203 | 0, -24, 30 | 5.13 | 0.048 |
| **Late extinction: CS+ > CS− (HC > Anxiety group)** | | | | | |
| No differential results |  |  |  |  |  |
| **Late extinction: HC > Anxiety group (across CSs)** | | | | | |
| Lingual gyrus (R+L) + calcarine (R+L) | R+L | 155 | 6, -88, 2 | 4.93 | 0.105 |
| Lobule VI of cerebellar hemisphere + fusiform gyrus | L | 111 | -30, -56, -16 | 4.32 | 0.221 |
| **Early Extinction CS+ > CS− vs. Late Extinction CS+ > CS− (across group)** | | | | | |
| Supramarginal gyrus + postcentral gyrus + lingual gyrus | L | 105 | -62, -22, 26 | 3.86 | 0.247 |
| **Early Extinction CS+ > CS− vs. Late Extinction CS+ > CS− (HC > Anxiety group)** | | | | | |
| No differential results |  |  |  |  |  |
| **Abbreviations:** CS+: conditioned fearful stimulus; CS−: conditioned safety stimulus; HC: healthy controls; L: left; MNI: Montreal Neurological Institute; no. voxel: number of voxels per cluster; R: right. | | | | | |

| **Table 3.** Whole-brain analysis results: clusters showing the linear trend analysis across EX1-EX4 [3 1 -1 -3], probed by the delayed fear extinction fMRI paradigm | | | | | |
| --- | --- | --- | --- | --- | --- |
| **Contrast and Cluster Region** | **Side** | **Cluster Size**  **(voxels)** | **Peak MNI Coordinates**  **(x, y, z)** | **Z-value** | **Cluster p**  **(FWE-corr)** |
| **[(EX1 > EX4) HC − (EX1 > EX4) Anxiety group], across CSs** | | | | | |
| Rolandic operculum + supramarginal gyrus + **insula** | R | 138 | 44, -16, 24 | 4.74 | 0.163 |
| **[(EX1 > EX4) Anxiety group − (EX1 > EX4) HC], across CSs** | | | | | |
| No differential results |  |  |  |  |  |
| **[(CS+ EX1 > CS+ EX4) − (CS− EX1 > CS− EX4)], across groups** | | | | | |
| Rolandic operculum + supramarginal gyrus + superior temporal gyrus + postcentral gyrus + insula + postcentral gyrus | R | 767 | 44, -22, 24 | 5.50 | 0.000 |
| Supramarginal gyrus + postcentral gyrus + superior temporal gyrus | L | 486 | -54, -24, 26 | 5.27 | 0.002 |
| Supplementary motor cortex (R+L) + middle cingulate cortex (R+L) + superior frontal gyrus (R) + anterior cingulate cortex, supracallosal (R+L) | R+ L | 1042 | 4, -2, 38 | 4.39 | 0.000 |
| **[(CS− EX1 > CS− EX4) − (CS+ EX1 > CS+ EX4)], across groups** | | | | | |
| No differential results |  |  |  |  |  |
| **Group (HC vs. Anxiety group) × Stimulus type (CS+ vs. CS−) interaction on attenuation (EX1 > EX4)** | | | | | |
| No differential results |  |  |  |  |  |
| **Abbreviations:** CS+: conditioned fearful stimulus; CS−: conditioned safety stimulus; CSs: CS+ and CS−; HC: healthy controls; L: left; MNI: Montreal Neurological Institute; no. voxel: number of voxels per cluster; R: right. | | | | | |

| **Table 4.** Whole-brain analysis results: clusters showing the attenuation of neural activation (EX1 > EX4) by group, probed by the delayed fear extinction fMRI paradigm | | | | | |
| --- | --- | --- | --- | --- | --- |
| **Contrast and Cluster Region** | **Side** | **no. voxels** | **MNI coordinates**  **peak voxel**  **(x, y, z)** | **Z-value** | **Cluster p**  **(FWE-corr)** |
| **HC: Early Extinction (EX1) > Late Extinction (EX4) across CSs** | | | | | |
| Insula (R+L) + supramarginal gyrus (R+L)+ calcarine (R+L) + lingual gyrus (R+L) + rolandic operculum (R+L) + putamen (R+L) + cuneus (R+L) + precentral gyrus (R+L) + superior temporal gyrus (R+L) + fusiform (R+L) + middle frontal gyrus (R+L) + superior temporal gyrus (R+L) + inferior frontal gyrus (R+L) + postcentral gyrus (R+L) + caudate (R+L) + precuneus (R+L) + middle temporal gyrus (R+L) + IFG pars orbitalis (R+L) + angular gyrus (R+L) + ventral anterior (R+L) + mediodorsal medial magnocellular (R+L) + ventral posterolateral (R+L) + cerebellum (R+L) + parahippocampal gyrus (R+L) + heschl’s gyrus (R+L) + amygdala (R+L) + superior frontal gyrus (R+L) | R+L | 21927 | -32, 24, 6 | Inf | 0.000 |
| Supplementary motor area (R+L) + middle cingulate cortex (R+L) + superior frontal gyrus (R+L) + anterior cingulate cortex, pregenual (R+L) + anterior cingulate cortex, supracallosal (R+L) + precentral gyrus (R+L) | R+L | 5817 | 6, 10, 48 | Inf | 0.000 |
| Precentral gyrus + postcentral gyrus + middle frontal gyrus | L | 821 | -38, -4, 48 | Inf | 0.000 |
| Middle frontal gyrus + inferior frontal gyrus, triangular part | R | 177 | 36, 34, 28 | 4.36 | 0.083 |
| **Anxiety group: Early Extinction (EX1) > Late Extinction (EX4) across CSs** | | | | | |
| Insula + inferior frontal gyrus, opercular part + inferior frontal gyrus, triangular part + precentral gyrus + rolandic operculum + middle frontal gyrus + putamen + IFG pars orbitalis + superior temporal gyrus + superior frontal gyrus + amygdala | R | 3972 | 34, 22, -2 | Inf | 0.000 |
| Insula + inferior frontal gyrus, triangular part + inferior frontal gyrus, opercular part + middle frontal gyrus + rolandic operculum + IFG pars orbitalis + superior temporal gyrus + precentral gyrus + putamen + superior frontal gyrus + heschl gyrus + postcentral gyrus + amygdala | L | 4363 | -32, 22, 4 | Inf | 0.000 |
| Supplementary motor area (R+L) + superior frontal gyrus, medial (R+L) + middle cingulate cortex (R+L) + anterior cingulate cortex, supracallosal (R+L) + anterior cingulate cortex, pregenual (R+L) | R+L | 4783 | 2,10, 38 | Inf | 0.000 |
| Ventral lateral (R+L) + caudate (R+L) + ventral anterior (R+L) + mediodorsal medial magnocellular + pulvinar medial (R+L) + putamen (R+L) + mediodorsal lateral parvocellular (R+L) + ventral posterolateral (R+L) + lateral posterior (R+L) + pulvinar lateral (R+L) + pulvinar inferior (R+L) lenticular nucleus, pallidum (R+L) | R+L | 2120 | 10, 2, 2 | Inf | 0.000 |
| Fusiform + lingual gyrus + inferior occipital gyrus + cerebelum + middle occipital gyrus + inferior temporal gyrus + calcarine + para hippocampal gyrus + precuneus + middle temporal gyrus + cerebelum | R | 1919 | 36, -58, -14 | 7.73 | 0.000 |
| Cerebelum + fusiform + lingual gyrus + calcarine + vermis + inferior occipital gyrus + inferior temporal gyrus + precuneus + parahippocampal gyrus | L | 1604 | -34, -58, -14 | 6.84 | 0.000 |
| Supramarginal gyrus + rolandic operculum + superior temporal gyrus + postcentral gyrus + angular gyrus + middle temporal gyrus | L | 962 | 64, -22, 22 | 5.77 | 0.000 |
| Supramarginal gyrus + inferior parietal gyrus + angular gyrus + superior temporal gyrus + postcentral gyrus + middle temporal gyrus | L | 975 | -56, -42, 30 | 5,73 | 0.000 |
| Precentral gyrus + middle frontal gyrus | L | 305 | -40, 0, 56 | 5.17 | 0.013 |
| Middle frontal gyrus + superior frontal gyrus | R | 159 | 30, 44, 22 | 4.77 | 0.110 |
| Middle temporal gyrus | L | 182 | -58, -56, 10 | 4.27 | 0.077 |
| Middle temporal gyrus | R | 277 | 54, -36, -2 | 4.19 | 0.019 |
| **Abbreviations:** CS+: conditioned fearful stimulus; CS−: conditioned safety stimulus; HC: healthy controls; L: left; MNI: Montreal Neurological Institute; no. voxel: number of voxels per cluster; R: right. | | | | | |

| **Table 5.** Repeated Measures of ANOVA for US-expectancy | | | | | | |
| --- | --- | --- | --- | --- | --- | --- |
| **Source of variation** | **Sum of Squares** | **df** | **Mean Square** | **F** | **p** | **η²** |
| **Between subjects** |  |  |  |  |  |  |
| Time | 60018.93 | 2 | 30009.46 | 128.15 | <0.001 | 0.029 |
| Stimulus type | 817286.83 | 1 | 817286.83 | 708.59 | <0.001 | 0.393 |
| Time* Group | 1396.12 | 2 | 698.06 | 2.98 | 0.051 | 6.715×10-^4^ |
| Stimulus type* Group | 3163.22 | 1 | 3163.22 | 2.74 | 0.09 | 0.002 |
| Time* Stimulus type | 12458.49 | 2 | 6229.24 | 31.95 | < .001 | 0.006 |
| Time* Stimulus type* Group | 513.67 | 2 | 256.83 | 1.31 | 0.26 | 2.471×10-^4^ |
|  |  |  |  |  |  |  |
| **Within subjects** |  |  |  |  |  |  |
| Group | 5460.28 | 1 | 5460.28 | 4.82 | <0.05 | 0.003 |

| **Table 6.** Repeated Measures of ANOVA for Arousal | | | | | | |
| --- | --- | --- | --- | --- | --- | --- |
| **Source of variation** | **Sum of Squares** | **df** | **Mean Square** | **F** | **p** | **η²** |
| **Between subjects** |  |  |  |  |  |  |
| Time | 44168.70 | 2 | 22084.35 | 79.36 | < .001 | 0.025 |
| Stimulus type | 240066.50 | 1 | 240066.50 | 276.81 | < .001 | 0.139 |
| Time* Group | 18.04 | 2 | 9.02 | 0.03 | 0.96 | 1.041×10-^5^ |
| Stimulus type* Group | 183.22 | 1 | 183.22 | 0.21 | 0.64 | 1.058×10-^4^ |
| Time* Stimulus type | 2325.062 | 2 | 1162.53 | 6.43 | <0.01 | 0.001 |
| Time* Stimulus type* Group | 99.06 | 2 | 49.53 | 0.27 | 0.76 | 5.718×10-^5^ |
|  |  |  |  |  |  |  |
| **Within subjects** |  |  |  |  |  |  |
| Group | 47158.09 | 1 | 47158.09 | 24.26 | < .001 | 0.027 |

| **Table 7**. Repeated Measures of ANOVA for Valence | | | | | | |
| --- | --- | --- | --- | --- | --- | --- |
| **Source of variation** | **Sum of Squares** | **df** | **Mean Square** | **F** | **p** | **η²** |
| **Between subjects** |  |  |  |  |  |  |
| Time | 4609.92 | 2 | 2304.96 | 16.63 | < .001 | 0.006 |
| Stimulus type | 64222.61 | 1 | 64222.61 | 111.59 | < .001 | 0.09 |
| Time* Group | 170.84 | 2 | 85.42 | 0.61 | 0.54 | 2.399×10^-4^ |
| Stimulus type* Group | 1099.53 | 1 | 1099.53 | 1.911 | 0.16 | 0.002 |
| Time* Stimulus type | 735.68 | 2 | 367.84 | 3.20 | <0.05 | 0.001 |
| Time* Stimulus type* Group | 736.21 | 2 | 368.10 | 3.20 | <0.05 | 0.001 |
|  |  |  |  |  |  |  |
| **Within subjects** |  |  |  |  |  |  |
| Group | 6924.34 | 1 | 6924.34 | 11.39 | < .001 | 0.01 |

| **Table 8.** Whole-brain analysis results: clusters showing the changes in activation reduction from Early Extinction to Late Extinction (Early Extinction > Late Extinction), controlling for state anxiety, probed by the delayed fear extinction fMRI paradigm | | | | | |
| --- | --- | --- | --- | --- | --- |
| **Contrast and Cluster Region** | **Side** | **no. voxels** | **MNI coordinates**  **peak voxel**  **(x, y, z)** | **Z-value** | **Cluster p**  **(FWE-corr)** |
| **[(EX1 > EX4) HC − (EX1 > EX4) Anxiety group], across CSs** | | | | | |
| Rolandic operculum + superior temporal gyrus | R | 368 | 44, -16, 24 | 5.21 | 0.005 |
| Supplementary motor area (R+L) +middle cingulate & paracingulate gyri (L) | L+R | 269 | -6, 8, 46 | 4.45 | 0.020 |
| Precentral gyrus + postcentral gyrus | L | 225 | -38, -2, 48 | 4.42 | 0.037 |
| Postcentral gyrus + supramarginal gyrus + rolandic operculum | L | 233 | -62, -22, 20 | 3.77 | 0.033 |
| **[(EX1 > EX4) Anxiety group − (EX1 > EX4) HC], across CSs** | | | | | |
| No differential results |  |  |  |  |  |
| **[(CS+ EX1 > CS+ EX4) − (CS− EX1 > CS− EX4)], across groups** | | | | | |
| Superior temporal gyrus + postcentrl gyrus + superior temporal gyrus | L | 125 | -60, -22, 26 | 3.89 | 0.180 |
| Rolandic operculum + supramarginal gyrus | R | 107 | 44, -22, 24 | 3.80 | 0.244 |
| **[(CS− EX1 > CS− EX4) − (CS+ EX1 > CS+ EX4)], across groups** | | | | | |
| No differential results |  |  |  |  |  |
| **Group (HC vs. Anxiety group) × Stimulus type (CS+ vs. CS−) interaction on attenuation (EX1 > EX4)** | | | | | |
| No differential results |  |  |  |  |  |
| **Abbreviations:** CS+: conditioned fearful stimulus; CS−: conditioned safety stimulus; CSs: CS+ and CS−; HC: healthy controls; L: left; MNI: Montreal Neurological Institute; no. voxel: number of voxels per cluster; R: right. | | | | | |

**References**

1	Heinig I, Pittig A, Richter J, Hummel K, Alt I, Dickhöver K *et al.* Optimizing exposure-based CBT for anxiety disorders via enhanced extinction: Design and methods of a multicentre randomized clinical trial. *Int J Methods Psychiatr Res* 2017; **26**: e1560.

2	Pittig A, Heinig I, Goerigk S, Thiel F, Hummel K, Scholl L *et al.* Efficacy of temporally intensified exposure for anxiety disorders: A multicenter randomized clinical trial. *Depress Anxiety* 2021; **38**: 1169–1181.

3	Pittig A, Heinig I, Goerigk S, Richter J, Hollandt M, Lueken U *et al.* Change of Threat Expectancy as Mechanism of Exposure-Based Psychotherapy for Anxiety Disorders: Evidence From 8,484 Exposure Exercises of 605 Patients. *Clin Psychol Sci* 2023; **11**: 199–217.

4	Hollandt M, Wroblewski A, Yang Y, Ridderbusch IC, Kircher T, Hamm AO *et al.* Facilitating translational science in anxiety disorders by adjusting extinction training in the laboratory to exposure-based therapy procedures. *Transl Psychiatry* 2020; **10**: 110.

5	Ridderbusch IC, Wroblewski A, Yang Y, Richter J, Hollandt M, Hamm AO *et al.* Neural adaptation of cingulate and insular activity during delayed fear extinction: A replicable pattern across assessment sites and repeated measurements. *NeuroImage* 2021; **237**: 118157.

6	Wroblewski A, Hollandt M, Yang Y, Ridderbusch IC, Pietzner A, Szeska C *et al.* Sometimes I feel the fear of uncertainty stinging clear: How Intolerance of Uncertainty and Trait Anxiety impact fear acquisition, extinction and the return of fear. PsyArXiv, 2022 doi:10.31234/osf.io/pm9z5.

7	Wroblewski A, Hollandt M, Yang Y, Ridderbusch IC, Pietzner A, Szeska C *et al.* Sometimes I feel the fear of uncertainty: How intolerance of uncertainty and trait anxiety impact fear acquisition, extinction and the return of fear. *Int J Psychophysiol* 2022; **181**: 125–140.

8	Friedman L, Glover GH, Krenz D, Magnotta V. Reducing inter-scanner variability of activation in a multicenter fMRI study: Role of smoothness equalization. *NeuroImage* 2006; **32**: 1656–1668.

9	Stöcker T, Schneider F, Klein M, Habel U, Kellermann T, Zilles K *et al.* Automated quality assurance routines for fMRI data applied to a multicenter study. *Hum Brain Mapp* 2005; **25**: 237–246.

10	Slotnick SD. Cluster success: fMRI inferences for spatial extent have acceptable false-positive rates. *Cogn Neurosci* 2017; **8**: 150–155.
